# Clonal Expansion Analysis of Transposon Insertions by High-Throughput Sequencing Identifies Candidate Cancer Genes in a PiggyBac Mutagenesis Screen

**DOI:** 10.1371/journal.pone.0072338

**Published:** 2013-08-05

**Authors:** Roland H. Friedel, Caroline C. Friedel, Thomas Bonfert, Ruijin Shi, Roland Rad, Philippe Soriano

**Affiliations:** 1 Department of Neuroscience, Department of Developmental and Regenerative Biology, Department of Neurosurgery, Icahn School of Medicine at Mount, Sinai, New York, New York, United States of America; 2 Institute for Informatics, Ludwig-Maximilians-Universität München, Munich, Germany; 3 Department of Pathology, Department of Neurology, Icahn School of Medicine at Mount, Sinai, New York, New York, United States of America; 4 Wellcome Trust Sanger Institute, Genome Campus, Hinxton, United Kingdom; 5 Department of Developmental and Regenerative Biology, Department of Oncological Sciences, Tisch Cancer Institute, Icahn School of Medicine at Mount Sinai, New York, New York, United States of America; Southern Illinois University School of Medicine, United States of America

## Abstract

Somatic transposon mutagenesis in mice is an efficient strategy to investigate the genetic mechanisms of tumorigenesis. The identification of tumor driving transposon insertions traditionally requires the generation of large tumor cohorts to obtain information about common insertion sites. Tumor driving insertions are also characterized by their clonal expansion in tumor tissue, a phenomenon that is facilitated by the slow and evolving transformation process of transposon mutagenesis. We describe here an improved approach for the detection of tumor driving insertions that assesses the clonal expansion of insertions by quantifying the relative proportion of sequence reads obtained in individual tumors. To this end, we have developed a protocol for insertion site sequencing that utilizes acoustic shearing of tumor DNA and Illumina sequencing. We analyzed various solid tumors generated by PiggyBac mutagenesis and for each tumor >10^6^ reads corresponding to >10^4^ insertion sites were obtained. In each tumor, 9 to 25 insertions stood out by their enriched sequence read frequencies when compared to frequencies obtained from tail DNA controls. These enriched insertions are potential clonally expanded tumor driving insertions, and thus identify candidate cancer genes. The candidate cancer genes of our study comprised many established cancer genes, but also novel candidate genes such as Mastermind-like1 (*Mamld1*) and Diacylglycerolkinase delta (*Dgkd*). We show that clonal expansion analysis by high-throughput sequencing is a robust approach for the identification of candidate cancer genes in insertional mutagenesis screens on the level of individual tumors.

## Introduction

Somatic mutagenesis by DNA transposons in mice investigates the underlying genetics of tumorigenesis. Transposons harboring gene-activating or gene-trapping cassettes can activate oncogenes or disrupt tumor suppressors, thereby driving tumor growth [1]. In addition to their role in discovering novel cancer genes, transposon mutagenesis also facilitates more detailed studies on genetic and cellular mechanisms of tumorigenesis by utilizing sensitizing background mutations and cell type-specific transposon activation [2]. Two transposon systems have been successfully used in cancer screens in the mouse: Sleeping Beauty [2–4] and PiggyBac [5,6]. Transposon insertion sites are identified by sequencing junction fragments of transposon ends and flanking genomic DNA. Tumor driving candidate cancer genes are then identified by common insertion site (CIS) analysis, a process of mapping insertions of multiple tumors and analysis of genes that are commonly hit in independent samples. CIS analysis has proven to be a successful approach for identification of candidate genes, however, it requires a considerable number of tumor samples (50-100 in most studies), and it delivers very little information about the mutation patterns in individual tumors.

An important characteristic of transformation by transposons is the continuous mobilization in and out of insertion sites. This mechanism facilitates the positive selection and clonal expansion of tumor-driving insertions during tumor evolution over neutral passenger insertions. Clonal expansion of transposon insertions can be assessed by analyzing the relative proportions of sequence reads obtained from insertions in individual tumors. However, previous transposon tumor screens have not utilized clonal expansion analysis, likely due to limitations in the number of sequence reads obtained by the 454 Pyrosequencing approach used in these studies. In addition, standard protocols for tumor DNA preparation involve fragmentation by restriction digests, which introduces biases for insertions represented by shorter restriction fragments that are more efficiently amplified by PCR [7]. An alternative approach for DNA fragmentation, acoustic shearing, can significantly reduce PCR biases, as each insertion site is represented by a set of fragments with similar length range [8].

In order to obtain an improved quantitative representation of insertion sites by their sequence read numbers, we combined two technical advancements of recent reports – Illumina sequencing to obtain >10^6^ reads per sample [7], and acoustic shearing to reduce PCR biases [7,8] - and developed a protocol for efficient high-throughput sequencing of transposon insertions. We applied our protocol for the analysis of eleven various solid tumors that we had generated by mutagenesis with the PiggyBac transposon array ATP1-S2 [5]. For each insertion in each tumor, we calculated a relative read frequency, which enabled us to identify between 9–25 insertions per tumor representing clonally expanded tumor driving insertions. Among the genes mutated by clonally expanded insertions were numerous established cancer genes, and also several novel candidate cancer genes.

We demonstrate here that high-throughput sequencing of insertion site libraries identifies clonally expanded mutations in individual tumors. This approach will substantially improve the quality of candidate cancer gene predictions of insertional mutagenesis screens, and it will facilitate the identification of networks of cooperating mutations in individual tumors.

## Results

### PiggyBac transposon mutagenesis

**Figure 1:**
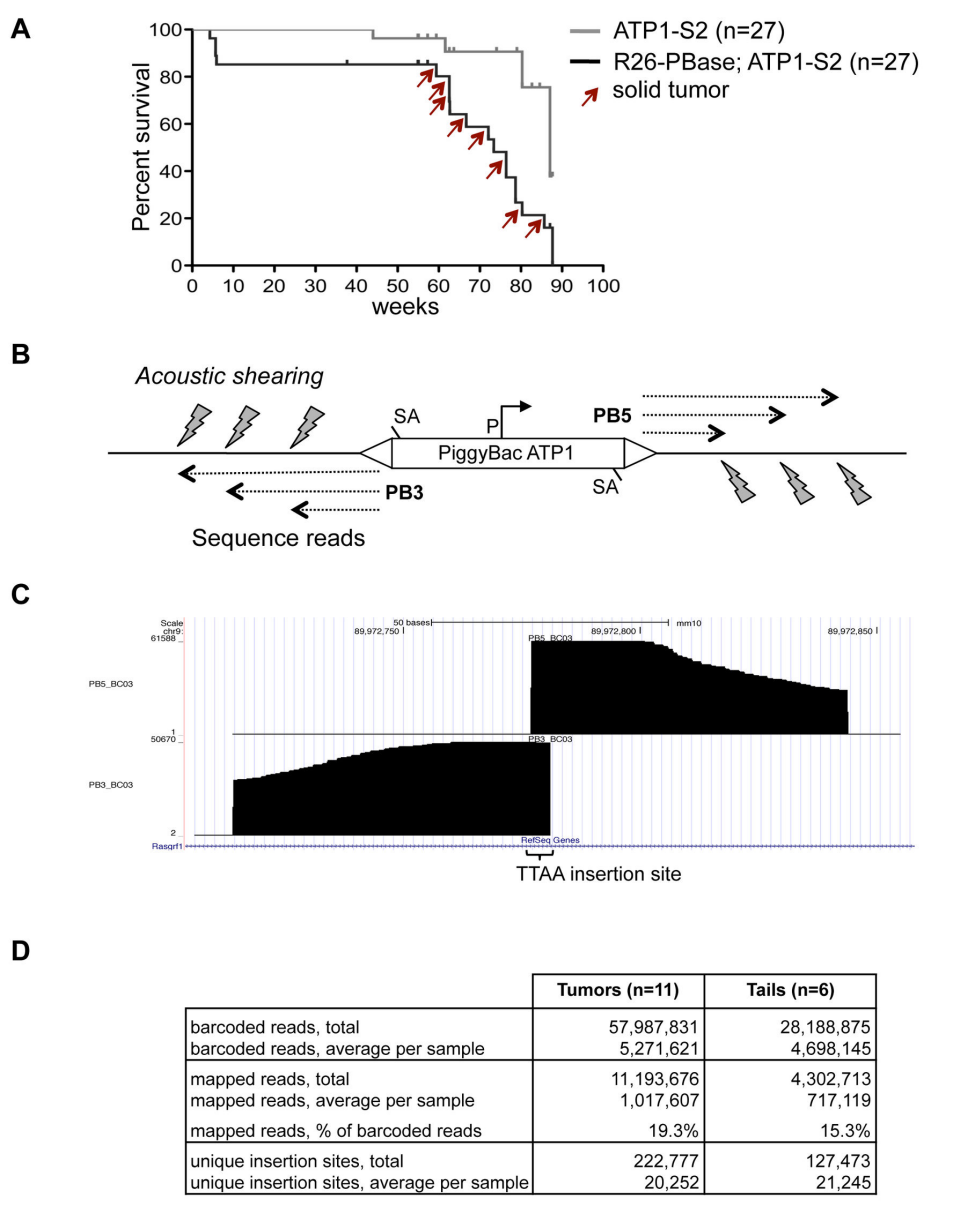
High-throughput sequencing of PiggyBac insertions. A) Kaplan-Meier survival plot of cohorts carrying either PiggyBac array alone (ATP1-S2) or PiggyBac array and constitutive transposase (ATP1-S2; R26-PBase). Red arrows indicate tumor occurrence. Tick marks denote censored animals (no tumor observed at the time of sacrifice). B) Alignments of Illumina genomic sequence reads terminate at positions generated by acoustic shearing. PB3/PB5, PiggyBac 3’/5’ terminal repeat; SA, splice acceptor; P, CAG promoter. C) Example of mapped sequence reads for the PB3 and PB5 sides of a PiggyBac insertion, viewed in the UCSC genome browser. Note that acoustic shearing causes random DNA break points to which Splinkerette adapters are ligated, which leads to a stair-like pattern of sequence alignments. The constant end of alignments to the right and left are caused by the invariable sequence read length of the Illumina system. D) Quantitative overview of sequence reads, mapped reads, and unique insertion sites from tail and tumor samples.

A transposon mutagenesis screen was performed in mice that carry a ubiquitously expressed PiggyBac transposase (ROSA26-PBase) and a transgenic PiggyBac transposon array (ATP1-S2) [5]. The ATP1-S2 array contains 20 copies of the transposon ATP1, which is equipped with splice acceptors for trapping of tumor suppressor genes and a CAG promoter for activation of oncogenes. These elements enable ATP1 to cause solid tumors upon transposition [5]. We bred a test cohort of 27 mice that carried ROSA26-PBase and ATP1-S2, and a control cohort of 27 mice with ATP1-S2 alone. Cohorts were aged, and while no tumors were observed in the control cohort, we observed 11 macroscopic tumors among 8 mice within the test cohort between 59 to 85 weeks (Figure 1A). Three animals carried tumors at two independent sites, and in one instance, genetic analysis of insertion sites indicated that both tumors share a common origin (see below), while all other tumors apparently arose independently. Tumor types comprised squamous cell carcinomas of the skin, solid tumors of lung and intestine, and follicular lymphoma of the spleen. The content of tumor cells in the dissected samples ranged from 30–100%, as assessed by histopathological analysis of hematoxylin and eosin stained sections (Figure S1). We isolated DNA for analysis of transposon insertion sites from all 11 tumors. To control for random insertions of PiggyBac in non-cancerous tissue, we isolated also DNA from 6 tail tips of tumor bearing mice.

### High-throughput sequencing of transposon insertions

We utilized Illumina high-throughput sequencing to achieve comprehensive read coverage of transposon insertions in individual tumor samples. In most previous transposon mutagenesis screens, tumor DNA was digested by restriction enzymes, which generate fragments of a constant size for individual insertions. As PCR amplifies shorter fragments more efficiently than longer fragments, a bias is introduced during the subsequent PCR amplification for insertions that are represented by short restriction fragments [7,8]. To achieve a more proportional representation of insertions, we fragmented tumor DNA by acoustic shearing into fragments of 200-400 bp size (see Methods S1 for a detailed protocol). Thereby, insertions are represented by fragment pools with similar size range. Shearing was followed by DNA end repair, A-tailing of 3’-ends, and ligation to Splinkerette adapters with 3’-T-overhang (Figure S2). In separate reactions, junction fragments for the 3’ and 5’ ends of the PiggyBac transposon (PB3 and PB5) were then amplified in two consecutive PCR rounds to generate PB3 and PB5 libraries for each sample. The PCR primers contained terminal adapter sequences for Illumina solid-phase amplification and sequencing and a 6-base bar code to distinguish samples in multiplex sequencing. We prepared two pools of PB3 and PB5 libraries, and each pool was sequenced on a single lane of an Illumina HiSeq2000 device with 100 bases read length. Sequence reads were demultiplexed according to their bar code and mapped to the mouse genome using the Bow tie algorithm [9].

Alignment of sequence reads in the UCSC genome browser confirmed the unbiased character of DNA fragmentation by acoustic shearing. The random distribution of DNA break points caused sequence reads from transposon insertions to align in a stair-like pattern around the central TTAA sequence of the PiggyBac insertion (Figure 1B, C). For each sample, we obtained on average ~5x10^6^ bar-coded reads, with similar numbers for tumors and tail controls (Figure 1D; Table S1).

About 19.3% of reads from tumor samples could be mapped to the mouse genome. For tail samples, 15.3% of reads could be mapped. These reads represent new insertions of the ATP1 transposon outside of its donor array. This moderate proportion of mappable reads was mainly due to a high fraction of reads that contained plasmid sequence flanking unmobilized transposons at the donor array and, thus, could not be mapped. To determine the fraction of ATP1 transposons that remain in the ATP1-S2 donor array, we performed Southern blot analysis of tails of ROSA26-PBase; ATP1-S2 mice and found that about 40% of ATP1-S2 transposons were not mobilized from the array (Figure S3). This incomplete mobilization of ATP1 may be caused by the specific in vivo chromatin status of the ATP1-S2 array. As DNA methylation at CpG sites can be inhibitory to PiggyBac transposition [10], we analyzed the methylation status of ATP1-S2 by Southern blot with a methylation sensitive restriction digest. CpG methylation of the ATP1-S2 array was detected, providing a possible explanation for the moderate transposition rate (Figure S3). An additional factor that reduces new transposon insertions is the loss of transposons during transposition (i.e., excision without following reintegration), and a previous study indicated that about half of all ATP1 transposon copies are lost during transposition activity [5]. Despite the moderate fraction of mappable sequence reads, our high-throughput sequencing protocol allowed us to recover significant numbers of reads for the aims of our study with ~1x10^6^ reads mapped on average for tumor samples and ~0.7x10^6^ reads for tail controls (Figure 1D).

### One-sided and two-sided read coverage of insertions

Altogether, we found 4,210 novel insertion sites in tumor and tail samples combined that were supported by read mappings on both sides of the PiggyBac transposon insertion (PB3 and PB5). Most insertions, however, were identified by mapped reads on only one side of the transposon (314,970 insertions with reads on only PB3 or PB5 side). Interestingly, the vast majority of insertions in our study, in particular those with reads mapped to only one side of the transposon, were represented by small read numbers (Figure S4). A similar observation about the high proportion of rare insertions has been made in a Sleeping Beauty study [7]. It is conceivable that these rare insertions escape detection on both sides of the transposon due to their low abundance, resulting in the high proportion of insertions covered on one side only. Interestingly, we also observed several insertions with high read numbers that were covered only on one side (Figure S4). Potential reasons for one-sided read coverage in these cases include flanking repetitive or low complexity DNA stretches that prevent mapping, or small DNA rearrangements (deletions, inversions) caused by the transposon insertion. For comprehensive analysis, we included in our study all insertion sites, combining insertion sites with one-sided and with two-sided sequence read coverage, which resulted in ~2x10^4^ unique transposon insertions on average per sample (Figure 1D).

### PiggyBac mutates the genome evenly

**Figure 2:**
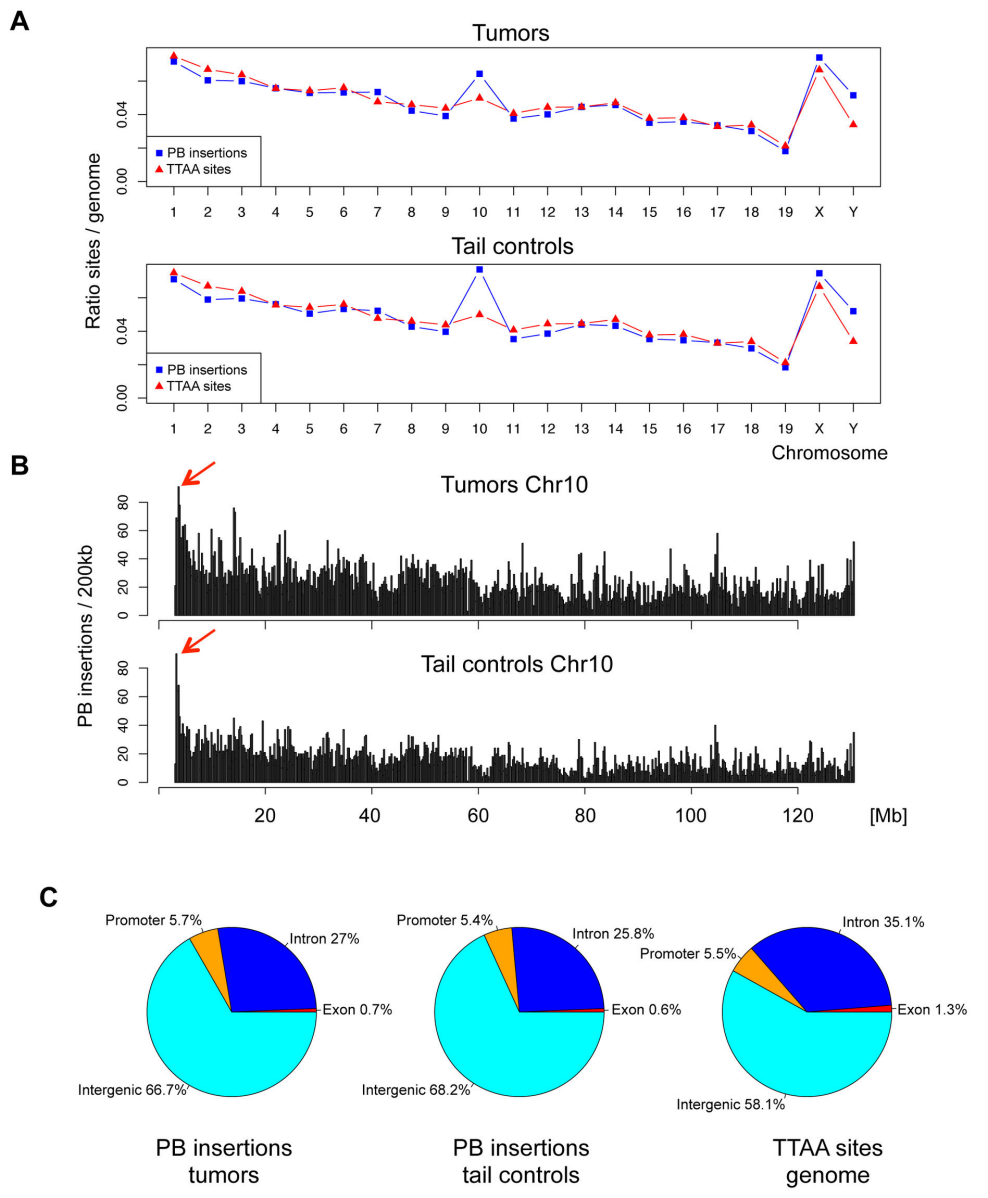
Genomic features of PiggyBac insertions in tumors and tail controls. A) Relative distribution of PiggyBac insertion sites over chromosomes, compared to proportion of TTAA sites on chromosomes. Note the overrepresentation of chromosome 10, which carries the transposon donor array. Chromosome Y, which consists mainly of highly repetitive elements, showed a slight preference for PiggyBac insertions. B) Local hopping effect on Chromosome 10. Histogram of insertion density on chromosome 10 reveals a positive bias for insertions at proximal end (arrows), where the ATP1-S2 donor array is located. C) Distribution of PiggyBac insertions in genomic regions: Promoter (10 kb upstream of TSS), exons, introns, and intergenic regions.

Data on the chromosomal distribution of ATP1 insertions revealed that the ATP1 PiggyBac transposon mutates chromosomes evenly in proportion to their TTAA content, both in tumors and tails (Figure 2A). This pattern is in agreement with previous data reported for the insertion pattern of the related ATP2 transposon [5]. The exception to the proportional distribution of insertions was the proximal end of chromosome 10, which harbors the ATP1-S2 donor array. Here, we observed an enrichment of insertions in the range of ~2 Mb at the proximal end of chromosome 10 (Figure 2B). This bias is likely caused by a local hopping effect of the PiggyBac transposon in the vicinity of the donor array, and insertions in this area were therefore excluded from further analysis.

We also compared the distribution of insertion sites in gene regions (promoter, exon, intron) and intergenic regions. Overall, both tail and tumor samples showed a distribution of insertions that resembled the average proportion of gene regions in the mouse genome, although PiggyBac insertions were observed in somewhat higher than expected numbers in intergenic regions (Figure 2C).

### Clonal expansion of transposon insertions

Our analysis of transposon insertions demonstrated no substantial differences in the insertion patterns of PiggyBac transposons in tumor and tail samples in terms of chromosome distribution and gene regions. We next analyzed the quantitative differences of sequence reads numbers for insertions in individual samples. The underlying assumption is that clonally expanded insertions are present in a majority of cells of a sample and should be represented by higher read numbers than neutral insertions that are only present in a smaller fraction of the sample.

To adjust for variations in the sequence read coverage of different samples, we first generated relative read frequencies by normalizing PB3 and PB5 side read numbers for the total number of reads from the respective sample library. As PB3 and PB5 read frequencies were derived from independently prepared PCR libraries, a perfect correlation between the PB3 and PB5 read frequencies could not be expected. Nonetheless, we observed a robust correlation between PB3 and PB5 read frequencies for PiggyBac insertions (Figure S5). To combine the information of read frequencies of the PB3 and PB5 sides of each insertion, we then calculated for each insertion the average read frequency from its PB3 and PB5 read frequencies. For insertions that were represented by reads on only one side, the average frequency equals 0.5 of the respective one-sided frequency.

**Figure 3:**
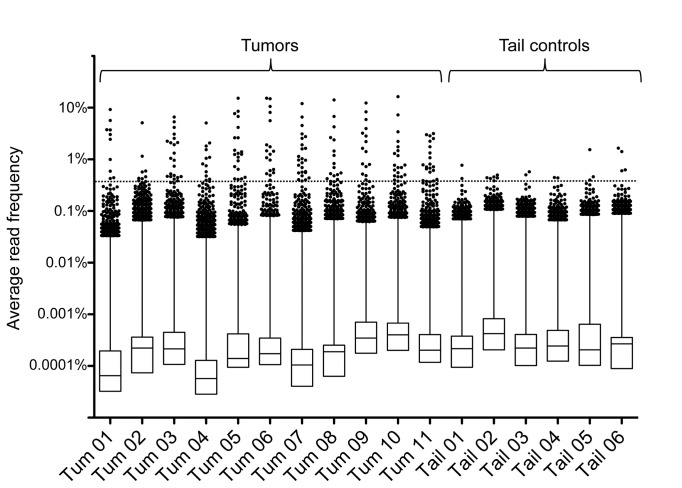
Tumors contain clonally expanded transposon insertions. Box plot of average read frequencies of transposon insertions in tumors and tail controls. Dots show the top 1% insertions of each sample, ranked by average read frequencies. Vertical line indicates distribution of the 2-25% percentile group, and boxes indicate distribution of the lower 75% of insertions. A threshold for outliers (dotted horizontal line) was calculated by averaging the read frequencies of the top 10 insertions of each tail sample.

For a comparative analysis of read frequency distributions in tumor and tail samples, we plotted the average read frequencies of all insertion sites in a box plot diagram (Figure 3). This diagram revealed that more than 99% of insertions in tumors and tails were represented by a small fraction of reads with read frequencies below 0.1%. Interestingly, however, when we focused on the top outliers of each sample, we observed that tumor samples contained a small number of outliers with enriched read frequencies that were clearly higher than the strongest outliers of tail samples. These high frequency insertions are likely to be clonally expanded insertions that have been positively selected for during tumor growth.

In absence of an efficient statistical method to distinguish true outliers from randomly enriched insertions, we decided to define clonal expansion by a threshold method. We utilized tail controls as a reference for the distribution of read frequencies in non-tumor tissue, and calculated a threshold for outlier detection by averaging the 60 highest frequencies of tail control samples (the top 10 frequencies of each tail sample). This resulted in a threshold of 0.37% for our dataset. We identified between 9–25 insertions for each tumor sample above threshold, but only 2-6 insertions in tail samples (Table S2).

We further interrogated the reproducibility of ranking insertion sites by read frequency in a series of control experiments. We first asked if insertion site expansion would vary between different parts of a single tumor mass (Figure S6). By comparing read frequencies of microdissected samples from single tumor masses, we observed a complete overlap of the most highly enriched insertions between samples and a match of more than 50% of insertions above threshold overall.

We then investigated if expanded insertions in tumors could already be present as expanded insertions in the pre-cancerous tissue from which the tumor arises. For this purpose, we compared transposon insertions into genes from a lung tumor with the insertions of adjacent normal lung tissue (Figure S7). The most highly enriched tumor insertions were not present in the normal tissue, confirming the validity of our approach to identify candidate cancer genes. However, 2 out of 6 expanded gene insertions of the tumor were also detected at similar levels in the normal lung tissue, indicating that some clonally expanded insertions in tumors can be derived from pre-cancerous clonal insertions.

Finally, we asked if organs would carry a higher background rate of expanded pre-cancer insertions than tail tissue, as organs have typically a more homogeneous composition of cell types than the heterogeneous tail (Figure S7). Indeed, we found that DNA from organs can in some cases contain more expanded insertions than tail tissue. The number of expanded insertions ranged from 3 to 23 in organs, with an average of 11 expanded insertions per sample. As the tumor samples of our cohort carried on average 16.4 expanded insertions, we estimate that on average 66% (11/16.4) of expanded insertions in tumors may reflect expanded pre-cancer insertions. Based on these control experiments, we conclude that clonal expansion analysis is an adequate method to identify candidate cancer genes in individual tumors. However, one important caveat is the presence of expanded pre-cancer insertions in tumor tissue, and the rate of these insertions may vary widely between samples depending on tumor type and host tissue.

Among clonally expanded insertions in tumors, 56.9% were found in gene regions (promoters, exons, and introns), -a significantly higher proportion than for all PiggyBac insertions in tumors (33.4%; see Figure 2C)-, and 43.1% were found in intergenic regions. Clonally expanded insertions in intergenic regions may exert long-range cis-effects on neighboring genes. However, as current databases do not contain information about the oncogenic role of intergenic regions, we limited our further analysis to transposon insertions in gene regions.

### Clonal expansions identify candidate cancer genes

**Figure 4:**
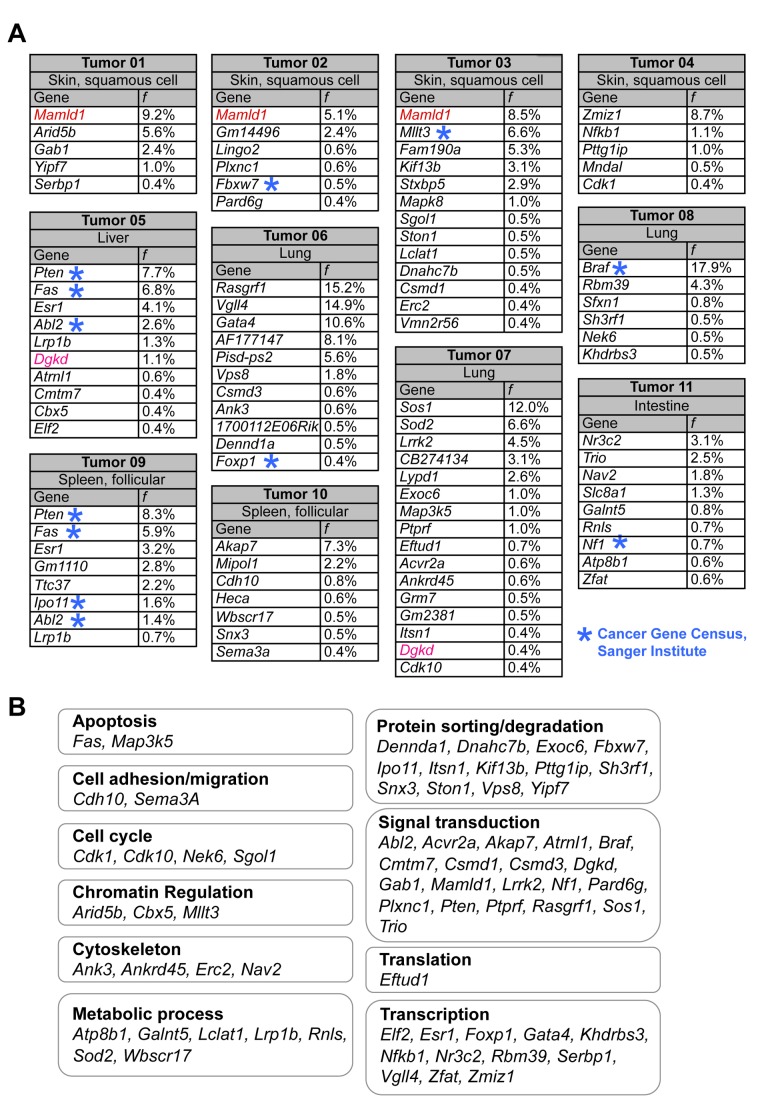
Genes associated with clonally expanded transposon insertions. A) Clonally expanded gene insertions in tumor samples, sorted by their average read frequencies (*f*). Three mice harbored two tumors each (tumor 01 and 08, tumor 05 and 09, and tumor 04 and 10, respectively). A blue asterisk denotes genes present in the Cancer Gene Census list, a list of 487 genes causally implicated in cancer. *Mamdl1* and *Dgkd* were hit in independent tumor samples and are highlighted in red and pink, respectively. B) Cellular processes affected by clonally expanded gene insertions. Process categories were compiled from functional gene information at www.omim.org and www.informatics.jax.org.

The clonal expansion analysis of transposon insertions in gene regions identified 88 unique genes that represent potential candidate cancer genes (Figure 4A). In agreement with this prediction, we observed several genes among our candidate genes that are implicated in cancer. For instance, nine of our candidate cancer genes were also contained in the Cancer Gene Census list, which comprises 487 genes causally linked to cancer [11], representing a highly significant enrichment (*Abl2*, *Braf*, *Fbxw7*, *Foxp1*, *Ipo11*, *Mllt3*, *Fas*, *Pten*, and *Nf1*; p<0.0002, Fisher’s exact test).

In our study, three cohort animals carried two tumors each (Figure S1). Interestingly, two pairs of tumors showed no overlap of candidate cancer genes (tumors 01 and 08; tumors 04 and 10), indicating independent origin of tumors. In contrast, tumors 05 (solid tumor mass in liver) and 09 (follicular lymphoma) shared 5 candidate cancer genes, indicating a common origin. As the liver is a common site of invasion for lymphocytic leukemia [12], it is possible that liver tumor 09 is a metastatic tumor mass that is derived from the splenic lymphocytic tumor 05. Of note, the 5 shared candidate cancer genes of tumors 05 and 09 (*Pten, Fas, Esr1, Abl2, Lrp1b*) were independently obtained in both tumors with similar average read frequencies, confirming the robustness of our sequencing method to identify clonal expansion of insertions.

When analyzing the candidate genes for significant enrichment of functional categories using the DAVID bioinformatics resource (http://david.abcc.ncifcrf.gov) [13], we found a significant enrichment of protein kinase related categories and pathways (e.g., KEGG: MAPK signaling pathway, p<0.016; INTERPRO: Protein kinase, core, p<0.016; Benjamini-Hochberg corrected P-value; Table S3). Likewise, when we manually grouped candidate genes by their known cellular process, we found that genes involved in signal transduction represented the largest group of genes (Figure 4B). Other prominent cellular processes of candidate genes were apoptosis, chromatin remodeling, transcription, and protein transport/sorting (Figure 4B). Interestingly, when looking at cellular processes on the level of individual tumors, we found that each tumor had one or several mutations in signaling genes, and a mix of insertions affecting at least two other cellular processes. This pattern of multiple mutated processes for each tumor is in agreement with the concept that multiple genetic alterations in different cellular processes drive tumor growth [14].

Among the candidate cancer genes defined by clonal expansion, two genes were hit independently at different TTAA sites in multiple tumors (excluding genes shared by tumor 05 and 09, which share a common origin): Mastermind-domain like 1 (*Mamld1*) and Diacylglycerolkinase delta (*Dgkd*). Thus, these genes are particularly strong candidate cancer genes, as they are doubly defined both by clonal expansion of insertions in individual tumors and independent occurrence as common insertion sites in multiple tumor samples. The *Mamld1* gene has been mutated by insertions in epithelial tumors of the skin (tumor 01, 02, and 03). *Mamld1* has been proposed to be a co-activator of non-canonical Notch signaling [15]. In human skin, Notch signaling components are abundantly expressed, and de-regulation of Notch signaling leads to various types of skin cancers [16]. Thus, *Mamld1* is a novel candidate cancer gene as a regulator of Notch signaling for epithelial tumors. The *Dgkd* gene has been found mutated in solid tumors 05 (lymphocytic metastasis in liver) and 07 (lung). *Dgkd* metabolizes 1,2, diacylglycerol (DAG) to phosphatidic acid (PA). Both DAG and PA play important roles as second messengers for mitogenic signals, and modulation of their levels by *Dgk* proteins has been proposed to be a potential mechanism in cancer [17].

## Discussion

We demonstrate here that high-throughput sequencing analysis of sheared DNA from transposon-generated tumors provides means to identify candidate cancer genes by clonal expansion analysis. This approach represents an improved strategy for the identification of candidate cancer genes in transposon screens, and allows for the first time the identification of candidate cancer genes at the level of individual tumors.

### Quantification of clonal expansion

Current protocols use restriction digests to fragment tumor DNA, resulting in a bias towards insertions that are represented by short fragments [7] and thereby reducing the quantitative aspect of read number analysis. We applied acoustic shearing for DNA fragmentation to minimize the size differences of insertion site fragments. This improvement, in combination with Illumina high-throughput sequencing, allowed us to obtain a semi-quantitative assessment of the proportional representation of insertions. Several observations suggest that our method reached a level of quantitative accuracy sufficient to identify clonally expanded insertions that define candidate cancer genes. First, the comparison of tumor and control samples revealed a distinct enrichment of insertion reads in tumor samples. Second, analysis of the related tumors 05 and 09 showed strong correlation of read frequencies of clonally expanded insertions. Finally, genes mutated by clonally expanded insertions comprised many well-defined cancer genes.

However, an important caveat of clonal expansion analysis is that organs can carry a number of pre-cancer insertions that are clonally expanded by chance. As our control experiments indicate, these insertions may comprise on average two-thirds of the clonally expanded insertions. Thus, although our method is clearly effective in identifying candidate cancer genes in individual tumors, the cancer driving function of each gene needs to be further corroborated by alterative methods, such as CIS analysis or functional assays.

A factor that can influence the clonal expansion rate of insertions is the activity of the PiggyBac transposase. If PiggyBac transposition activity would cease during the lifetime of the mouse, this would fix certain insertions, which would appear as clonal expansions in our analysis. We will address the continuous activity of the PiggyBac transposase in adult mouse tissue in future studies by immunohistochemical assays.

Several biases in sequence read representation in our clonal expansion analysis remain, for example those introduced by different GC content of fragments during PCR amplification. Another factor that decrease the quantitative power of our method are the potential presence of non-tumor stromal tissue in the samples, which dilutes the clonally expanded tumor driving insertions. Additionally, a caveat of our method is that non-specific Splinkerette primer binding could lead in some cases to a predominant amplification of genomic sites that are not true transposon insertions. Further technical refinements will be required to bring analysis of clonal expansion to an absolute quantitative level.

### High-throughput sequencing of transposon insertions

Our sequencing approach yielded >10^6^ mapped reads per tumor sample. A number of 10^5^ reads per sample had been proposed to be sufficient to reveal all insertions of Sleeping Beauty generated tumors [7]. In our data set, the vast majority of PiggyBac insertions were represented by low sequence read numbers, and it is reasonable to assume that with higher sequencing coverage an even higher number of rare insertions would be recovered. However, coverage of ~10^6^ reads per sample appears to be sufficient to identify clonally expanded transposon insertions.

Our screen revealed about 20,000 unique insertion sites per sample, both in tumors and in tail controls. In previous transposon screens with PiggyBac and Sleeping Beauty, which mainly analyzed restriction digest DNA libraries sequenced by 454 Pyrosequencing, the number of unique insertions per tumor sample ranged between 100–1000 (see, for example, 5,7,18–20). One possible reason for the higher number of insertions in our screen may be our improved protocol for the identification of insertion sites, which may result in a higher sensitivity of insertion site detection. Another reason may lie in the long lifetime of the ATP1-S2 tumor animals (70.5 weeks average), during which the transposon had more time to mobilize than in most previous screens, in which animals typically reached an age of 6-12 months.

Of note, previous studies on PiggyBac insertions had observed a bias of PiggyBac for insertion in gene regions [21–23]. Our data is not consistent with these studies, as we observed a slightly lower than expected rate of PiggyBac insertions in gene regions (see Figure 2C). A possible explanation for this discrepancy may lie in the high number of insertion sites sampled in our study (>100,000), which is more than two orders of magnitude higher than in previous studies.

As a consequence of the high numbers of insertion sites recovered by high throughput sequencing of tumors in transposon screens, the current unfiltered common insertion site (CIS) analysis approach generates long lists of candidate cancer genes. This is particularly obvious when considering that also non-cancerous tail tips carry high numbers of unique insertions. We therefore propose that future transposon tumor screens should analyze transposon insertions first by read frequency for their clonal expansion, and then limit CIS analyses to insertions that show a certain degree of clonal expansion.

### Networks of tumor driving mutations

The tumors in our study carried between 5–16 clonally expanded gene insertions. Such a number is in agreement with the classic prediction of 3-6 sequential mutations that are required to form a tumor [24]. A single transposon insertion may clearly not be enough to trigger a tumor, and the co-operation of multiple insertions in signaling networks and other cellular processes may be required. Our findings highlight the similarity of insertional transposon mutagenesis with the slow evolution of malignant tumors in human patients, confirming the suitability of the transposon system for cancer gene screens.

Interestingly, we did not find direct hits in components of the p53 and Rb tumor suppressor networks, which are the most frequent mutations in human tumors [25]. We do not know at present the status of these tumor suppressor pathways in our transposon mutagenesis tumors. Remarkably, however, we observed a strong bias among expanded insertions for signaling pathways, for example, downstream components of receptor tyrosine kinases signaling (*Braf, Gab1, Nf1, Pten, Sos1*), in agreement with data from human tumor studies that alterations in cellular signaling pathways are main drivers of tumorigenesis [14].

The clonal expansion analysis approach adds a new dimension to insertional tumor screens in the mouse, as it facilitates the identification of networks of cooperating mutations in individual tumors. The identification of cooperating mutations that drive tumorigenesis has important implications for our understanding of tumor biology and will eventually facilitate the design of combinatorial anti-cancer drug therapies.

## Materials and Methods

### Mouse lines

Animals were euthanized in accordance with National Institutes of Health Guidelines for the Care and Use of Laboratory Animals. The animal protocol was approved by IACUC committee at the Mount Sinai School of Medicine. The ATP1-S2 transgenic array line and the ROSA26-PiggyBac transposase line have been described previously [5]. Briefly, the ATP1-S2 line carries a transgenic array of 20 copies of the ATP1 transposon at the proximal end of chromosome 10, and the ROSA26-PBase is a knock-in of wildtype “insect” PiggyBac transposase into the ROSA26 gene locus. Tumor cohort mice were bred on a mixed 129/B6/Swiss genetic background. The Kaplan-Meier survival curve was generated using Prism 5 software (Graphpad Sofware, Inc.). Survival rates are calculated from animals that were found dead or were sacrificed because of an observable tumor or moribund appearance.

### Splinkerette PCR

DNA samples of tumors and tails were prepared for Illumina high throughput sequencing by acoustic shearing, end repair, A-tailing, and Splinkerette PCR based on published protocols [26,27]. The design of two sets of 17 bar-coded primers for the PB3 and PB5 sides of the second round of Splinkerette PCR was based on primers described in [7]. A detailed version of our Splinkerette protocol and all primer sequences can be found in Methods S1.

### Sequence analysis

Two DNA pools from Splinkerette products of 17 PB3 side and 17 PB5 side samples, respectively, were sequenced separately on single lanes of an Illumina HiSeq 2000 instrument with 100 base read lengths at the Genomics Core Facility, Mount Sinai School of Medicine. Samples were mixed before sequencing with 50% PhiX DNA library (Illumina) to increase cluster diversity on the sequencing chip.

After demultiplexing of bar-coded reads, PiggyBac transposon sequences were trimmed and remaining sequences were aligned to the mouse genome (GRCm38/mm10) using the Bow tie algorithm [9], with a seed size of 20 nucleotides, 1 allowed mismatch in the seed and any number of mismatches in the remaining read. Only the best alignment for each read was retained. In a second step, reads were trimmed so that the resulting alignment contained at most 4 mismatches. Reads that contained a starting position within a gamma satellite repeat element were filtered out, using annotations by RepeatMasker (http://www.repeatmasker.org).

Subsequently, reads were clustered according to their starting positions (corresponding to transposon insertion sites), and only starting positions and corresponding reads were retained that were supported by at least one read of a minimum size of *n* bases. To determine *n*, we used the fraction of reads from insertions that were supported by reads on both PB3 and PB5 sides as a guideline for assessing the quality of our sequence mappings (Figure S8). A minimum read length of *n*=34 was chosen for further analysis as it was the smallest cutoff value that provided for almost all samples high quality sequence mapping, while higher values of *n* provided no significant gain in mapping quality but lead to a decrease in the number of identified insertion sites. Normalized read frequencies were then calculated for every insertion site by dividing its associated read numbers by the total number of reads mapped in the sample.

Sequencing data of tumor and tail samples for the PB3 and PB5 side reads have been deposited at the NCBI Gene Expression Omnibus database (http://www.ncbi.nlm.nih.gov/geo/) under accession number GSE46210.

### Determination of clonal expansion of gene insertion

Insertion sites of each sample were sorted by their associated sequence read numbers, and insertions that were represented at identical positions in multiple samples with high read numbers were considered technical artifacts and removed from analysis. For each insertion site in each sample, an average read frequency was then calculated by averaging the normalized read frequencies of the PB3 and PB5 side reads (see Table S2). A threshold value was calculated by averaging the 60 highest average read frequencies of insertions in tail samples (10 from each tail). Clonally expanded gene insertions were defined by transposon insertions in gene regions (exons, introns, and 10 kb upstream) with average read frequencies higher than the threshold value.

## Supporting Information

Figure S1 PiggyBac tumor screen cohort. A) Age distribution and sites of tumor occurrence in R26-PBase; ATP1-S2 mice.
B) Tumors obtained in transposon screen. Upper panels show macroscopic appearance; lower panels are micrographs of hematoxylin and eosin stainings of the respective tumors. Tumor samples are characterized by high density of hematoxylin stained nuclei (purple). Scale bar in tumor 11 for all micrographs: 50 µm.
C) Histopathological analysis of hematoxylin and eosin stained sections of tumor tissues.
(PDF)

Figure S2 Preparation of insertion site libraries for Illumina sequencing. A) Steps for generation of insertion site library (see Methods S1 for details).
B) Scheme of the PB3 and PB5 side library pools that were sequenced on Illumina HiSeq 2000 chip. Sequence reads with Illumina standard sequencing primer begin with 6 bases bar code (BC01 through BC17), followed by PB3 (or PB5, respectively) transposon end and flanking genomic sequence.
(PDF)

Figure S3 Southern blot analysis of ATP1 transposition rate and methylation status of ATP1-S2 donor locus. A) A probe against the PB3 terminal repeat of ATP1 detects a repetitive band of the concatemeric ATP1-S2 donor array in *Bam*HI digested tail DNA (arrow). Mice carrying both ATP1-S2 transposon array and ROSA26-PBase show a reduction in band intensity due to transposition of ATP1 out of the array.
B) Densitometric quantification of array bands shown in A). Mice carrying transposon array and transposase reveal on average an array band intensity of 42% in comparison to the original donor array.
C) Tail DNA of mice carrying the ATP1-S2 transposon array was digested with MspI or the CpG methylation sensitive isoschizomer HpaII and probed for the PB3 terminal repeat. The upward shift of the repetitive array band in HpaII digests indicates presence of CpG methylation at the ATP1-S2 array.
(PDF)

Figure S4 Distribution of read numbers for transposon insertions. A) Distribution of read numbers for insertions that were covered by reads on both PB3 and PB5 sides (read numbers are sum of PB3 and PB5 side reads).
B) Distribution of read numbers for insertions that were covered by reads on one side only (either PB3 or PB5 side).
(PDF)

Figure S5 Correlation of PB3 and PB5 read frequencies. Clonally expanded transposon insertions as defined by threshold (see Figure 3) and with two-sided read coverage (151 insertions total) were plotted by PB5 and PB3 side read frequencies. The black line indicates linear regression analysis (slope 0.666 +/- 0.053; R^2^=0.30).
(PDF)

Figure S6 Correlation of PB5 read frequencies from different microdomains of a single tumor. A) Two pieces of a Formalin-stored sample of Tumor 05 were dissected and processed independently. Analysis of PiggyBac insertion sites and read frequencies were performed for the PB5 side of the transposon. Box plot diagram of read frequencies and tabular lists of enriched insertions above the 0.37% threshold show strong correlation between the samples. The 6 highest insertions rank in identical order, and more than 50% of enriched insertions are overall identical.
B) A lung tumor that had been obtained in a transposon mutagenesis mouse with additional heterozygous background mutation of the tumor suppressor INK4A/Arf [28] was dissected into two pieces that were processed independently for read frequencies from the PB5 side. Box plot diagram of read frequencies and tabular lists of enriched insertions above the 0.37% threshold show strong correlation, with more than 50% identical enriched insertions.
(PDF)

Figure S7 Comparison of insertion enrichment in tumor tissue, adjacent tissue, other organs, and tail tips. A) Transposon insertions in a lung tumor that had been obtained in a transposon mouse with INK4A/Arf^+/-^ background were analyzed for PB5 side read frequencies and compared to samples from adjacent normal lung tissue, liver, and tail of the same mouse. The tabular listings of enriched insertions in genes in these samples reveal that the lung tumor carries an expanded insertion in the Rasrgf1 gene, which was not obtained in other tissues. Rasgrf1 is therefore a strong candidate cancer gene. Of note, Rasgrf1 is upregulated in lung tumors [29], and Rasgrf1 is also the most enriched insertion site in the lung Tumor 06 (see Figure 4A). An expanded insertion in the Myod1 gene was also observed the tumor sample, which was found at about tenfold lower frequency in other tissues and tail. Expanded insertions in the *Reep3* and *Syn3* genes were found both in tumor and adjacent normal lung tissue at similar frequency, suggesting that these insertions are already present in normal tissue and are not tumor driving.
B) The tumor free transposon mutagenesis mouse #3805 was dissected, and PiggyBac insertion profiles of different organs and tails were compared. The number of expanded insertions above the 0.37% threshold is indicated in the diagram. Between 3 and 23 enriched insertions were found in organs, and 2 in tail tissue.
(PDF)

Figure S8 Read length cut-off for the identification of insertion sites. The fraction of reads from insertions with reads on both the PB3 and PB5 sides were plotted against different minimum read length values for identification of insertion sites. A value of *n*=34 was chosen for final analysis (see Methods for details). Note the significant higher fraction of reads for PB3/PB5 insertions sites in tumor (BC01-BC11) vs. tail samples (BC12-BC17).
(PDF)

Table S1 Sequence reads and insertions in individual samples. (XLSX)

Table S2 Clonally expanded insertions. (XLSX)

Table S3 DAVID analysis of candidate cancer genes defined by clonal expansion. (XLSX)

Methods S1 Detailed description of Splinkerette PCR protocol and oligonucleotide sequences. (PDF)
