# Supplementary material for: Clonal Expansion Analysis of Transposon Insertions by High-Throughput Sequencing Identifies Candidate Cancer Genes in a PiggyBac Mutagenesis Screen
Source: PLoS One. 2013 Aug 5;8(8):e72338. doi: 10.1371/journal.pone.0072338 (PMC3733837; doi:10.1371/journal.pone.0072338)
Supplement: Figure S1 — A) Age distribution and sites of tumor occurrence in R26-PBase; ATP1-S2 mice. B) Tumors obtained in transposon screen. Upper panels show macroscopic appearance; lower panels are micrographs of hematoxylin and eosin stainings of the respective tumors. Tumor samples are characterized by high density of hematoxylin stained nuclei (purple). Scale bar in tumor 11 for all micrographs: 50 µm. C) Histopathological analysis of hematoxylin and eosin stained sections of tumor tissues. (PDF) [file pone.0072338.s001.pdf]

**A**

| Animal ID | Age | Tumor sites | Tumor # | Tail control |
|---|---|---|---|---|
| #1267 | 59 wks | liver, spleen | 05, 09 | tail 01 |
| #1221 | 63 wks | lung | 06 | tail 02 |
| #1225 | 63 wks | skin | 03 | tail 03 |
| #1222 | 67 wks | intestines | 11 | |
| #1081 | 72 wks | lung | 07 | |
| #1218 | 76 wks | oral | 02 | tail 04 |
| #839 | 79 wks | oral, lung | 01, 08 | tail 05 |
| #1157 | 85 wks | skin, spleen | 04, 10 | tail 06 |

**B**

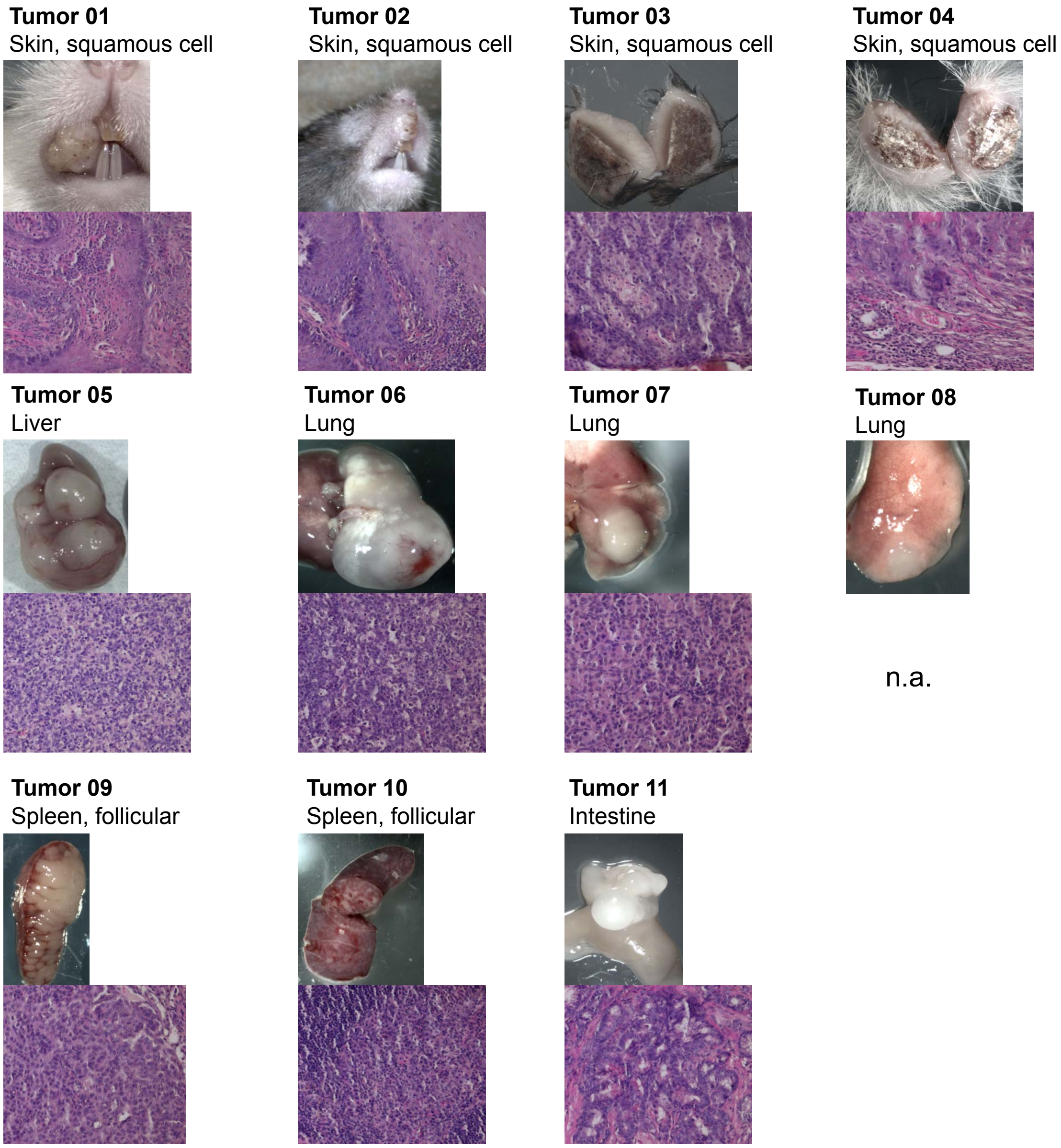


**C**

| Tumor# | location | tumor% | comments |
|---|---|---|---|
| 01 | skin | 95 | SCC with 5% stroma |
| 02 | skin | 90 | SCC with 10% stroma |
| 03 | skin | 85 | SCC with 15% stroma |
| 04 | skin | 30 | SCC with 60% keratin,10% stroma |
| 05 | liver | 100 | tumor cells similar as tumor# 9 |
| 06 | lung | 75 | adenocarcinoma like tumor with 25% adjacent normal lung |
| 07 | lung | 85 | adenocarcinoma like tumor with 15% adjacent normal lung |
| 08 | lung | n.a. | no tumor tissue for histology available |
| 09 | spleen | 100 | tumor cells similar as tumor# 5 |
| 10 | spleen | 100 | lymphoma? |
| 11 | intestine | 60 | adenocarcinoma like tumor with 5% adjacent normal mucosa, 20% stroma, 15% cyst. gland |
