# Supplementary material for: Clonal Expansion Analysis of Transposon Insertions by High-Throughput Sequencing Identifies Candidate Cancer Genes in a PiggyBac Mutagenesis Screen
Source: PLoS One. 2013 Aug 5;8(8):e72338. doi: 10.1371/journal.pone.0072338 (PMC3733837; doi:10.1371/journal.pone.0072338)
Supplement: Figure S2 — A) Steps for generation of insertion site library (see Methods S1 for details). B) Scheme of the PB3 and PB5 side library pools that were sequenced on Illumina HiSeq 2000 chip. Sequence reads with Illumina standard sequencing primer begin with 6 bases bar code (BC01 through BC17), followed by PB3 (or PB5, respectively) transposon end and flanking genomic sequence. (PDF) [file pone.0072338.s002.pdf]

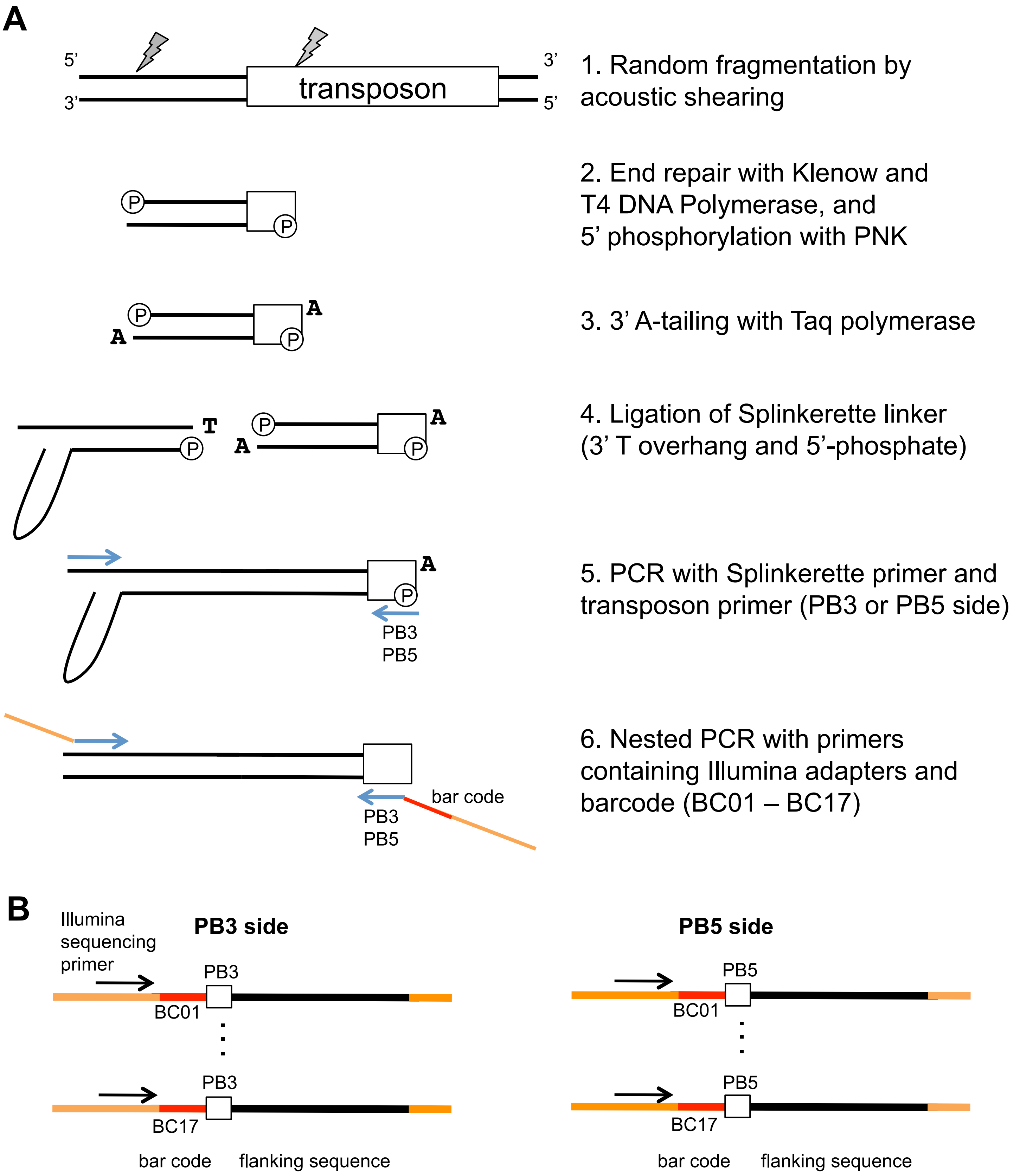
A
5'
3'
3'
5'
transposon
1. Random fragmentation by acoustic shearing
P
P
2. End repair with Klenow and T4 DNA Polymerase, and 5' phosphorylation with PNK
A
A
3. 3' A-tailing with Taq polymerase
T
A
A
4. Ligation of Splinkerette linker (3' T overhang and 5'-phosphate)
A
PB3
PB5
5. PCR with Splinkerette primer and transposon primer (PB3 or PB5 side)
bar code
PB3
PB5
6. Nested PCR with primers containing Illumina adapters and barcode (BC01 – BC17)
B
Illumina sequencing primer
PB3 side
PB3
BC01
PB3
BC17
bar code
flanking sequence
PB5 side
PB5
BC01
PB5
BC17
bar code
flanking sequence
