# Supplementary figures and images for: Clonal Expansion Analysis of Transposon Insertions by High-Throughput Sequencing Identifies Candidate Cancer Genes in a PiggyBac Mutagenesis Screen
Source: PLoS One. 2013 Aug 5;8(8):e72338. doi: 10.1371/journal.pone.0072338 (PMC3733837; doi:10.1371/journal.pone.0072338)

**A**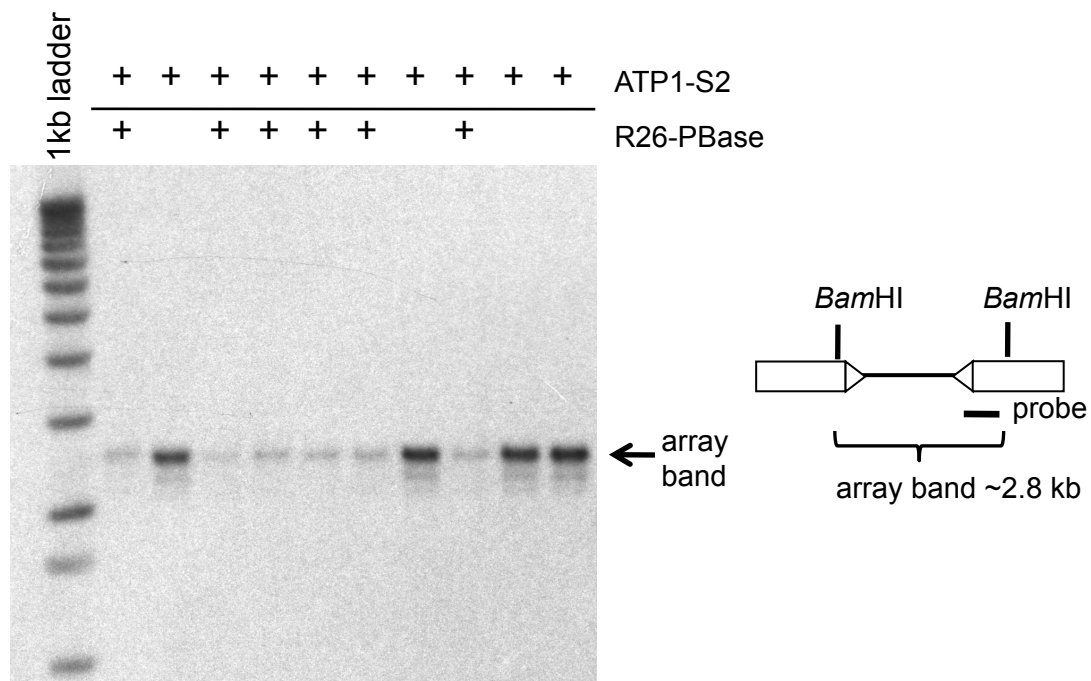**B**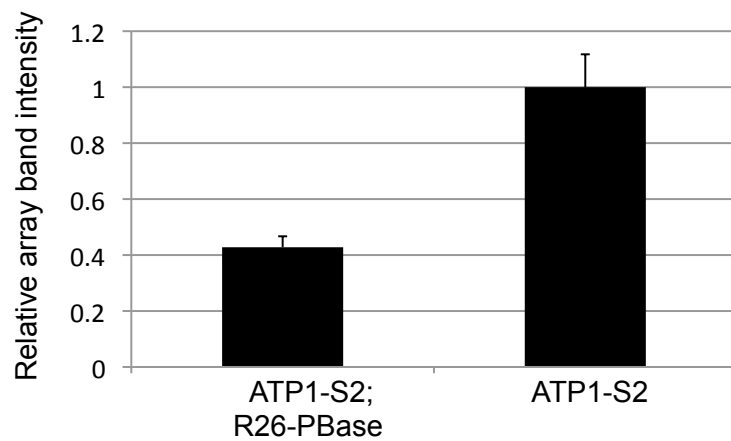**C**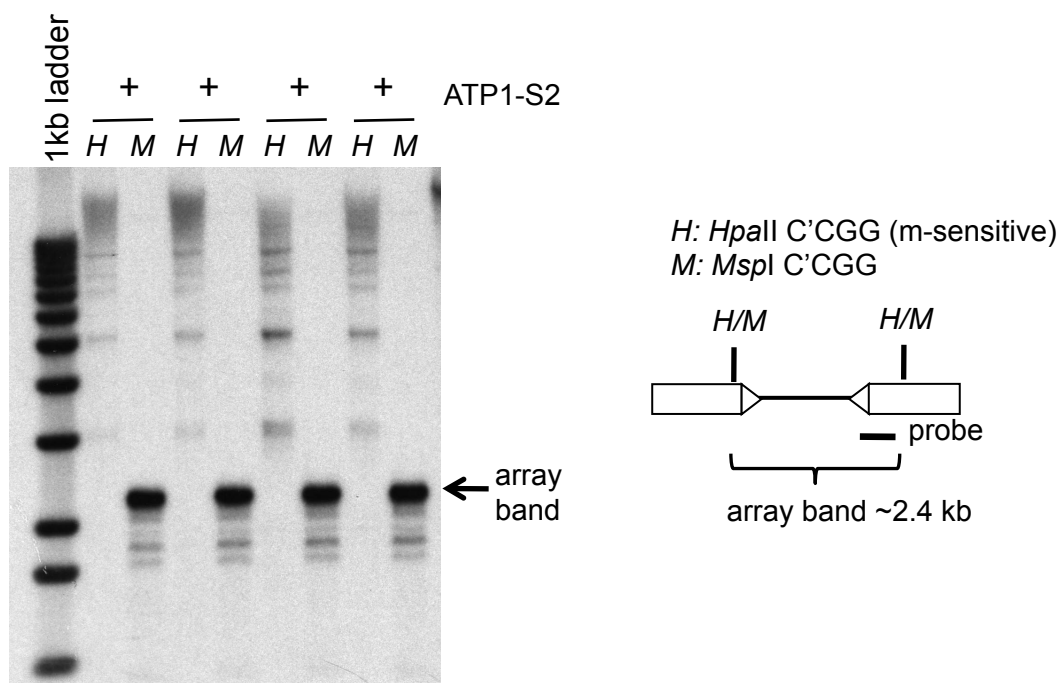

Supplement: Figure S3 — A) A probe against the PB3 terminal repeat of ATP1 detects a repetitive band of the concatemeric ATP1-S2 donor array in BamHI digested tail DNA (arrow). Mice carrying both ATP1-S2 transposon array and ROSA26-PBase show a reduction in band intensity due to transposition of ATP1 out of the array. B) Densitometric quantification of array bands shown in A). Mice carrying transposon array and transposase reveal on average an array band intensity of 42% in comparison to the original donor array. C) Tail DNA of mice carrying the ATP1-S2 transposon array was digested with MspI or the CpG methylation sensitive isoschizomer HpaII and probed for the PB3 terminal repeat. The upward shift of the repetitive array band in HpaII digests indicates presence of CpG methylation at the ATP1-S2 array. (PDF) [file pone.0072338.s003.pdf]

**A**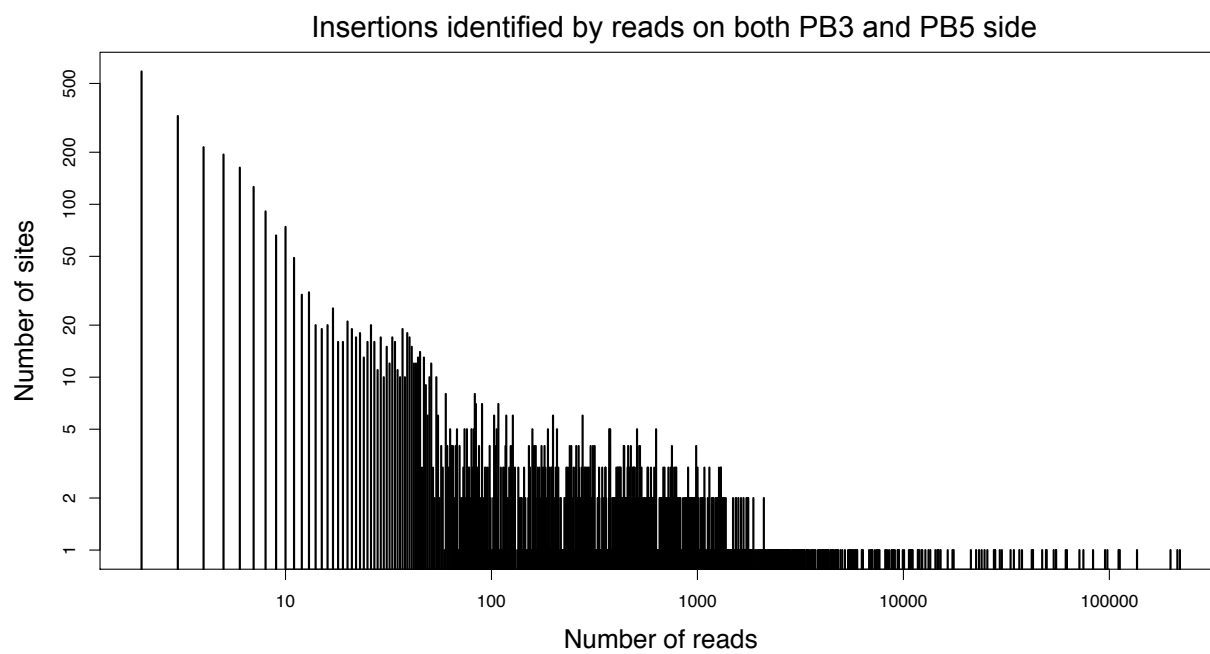**B**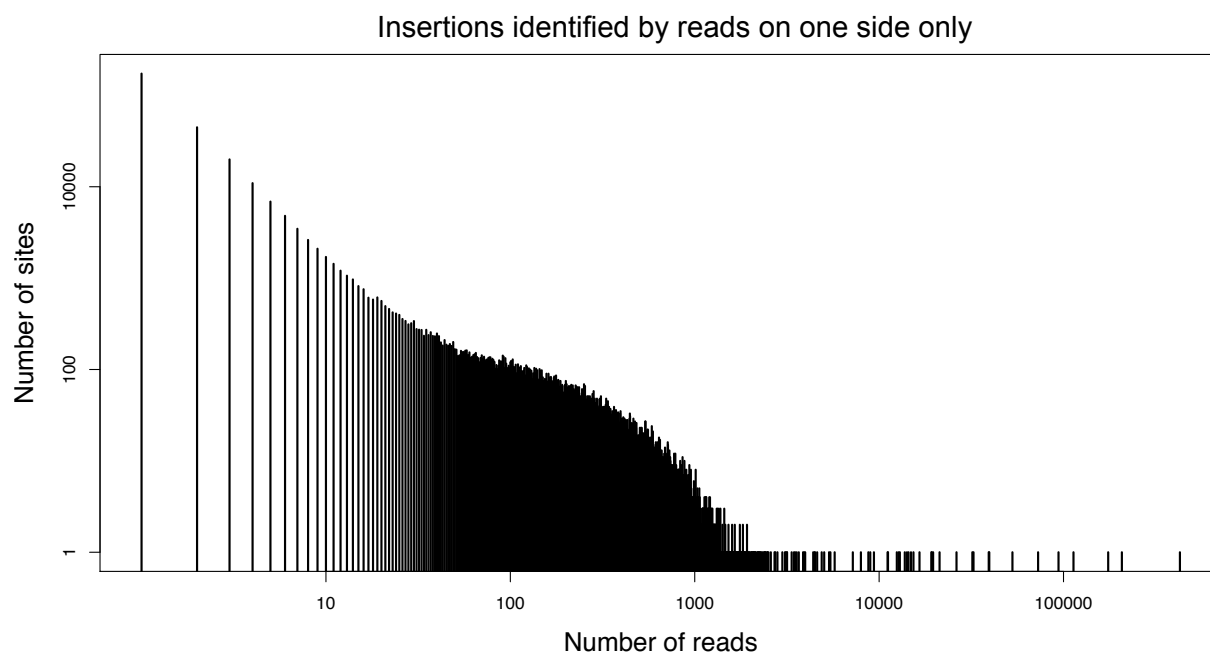

Supplement: Figure S4 — A) Distribution of read numbers for insertions that were covered by reads on both PB3 and PB5 sides (read numbers are sum of PB3 and PB5 side reads). B) Distribution of read numbers for insertions that were covered by reads on one side only (either PB3 or PB5 side). (PDF) [file pone.0072338.s004.pdf]

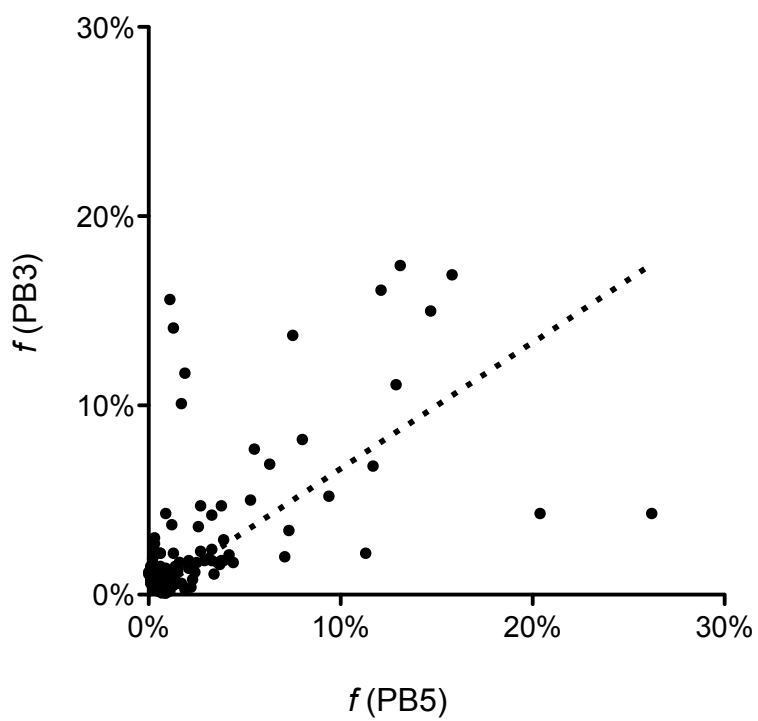

Supplement: Figure S5 — Clonally expanded transposon insertions as defined by threshold (see Figure 3) and with two-sided read coverage (151 insertions total) were plotted by PB5 and PB3 side read frequencies. The black line indicates linear regression analysis (slope 0.666 +/- 0.053; R2=0.30). (PDF) [file pone.0072338.s005.pdf]

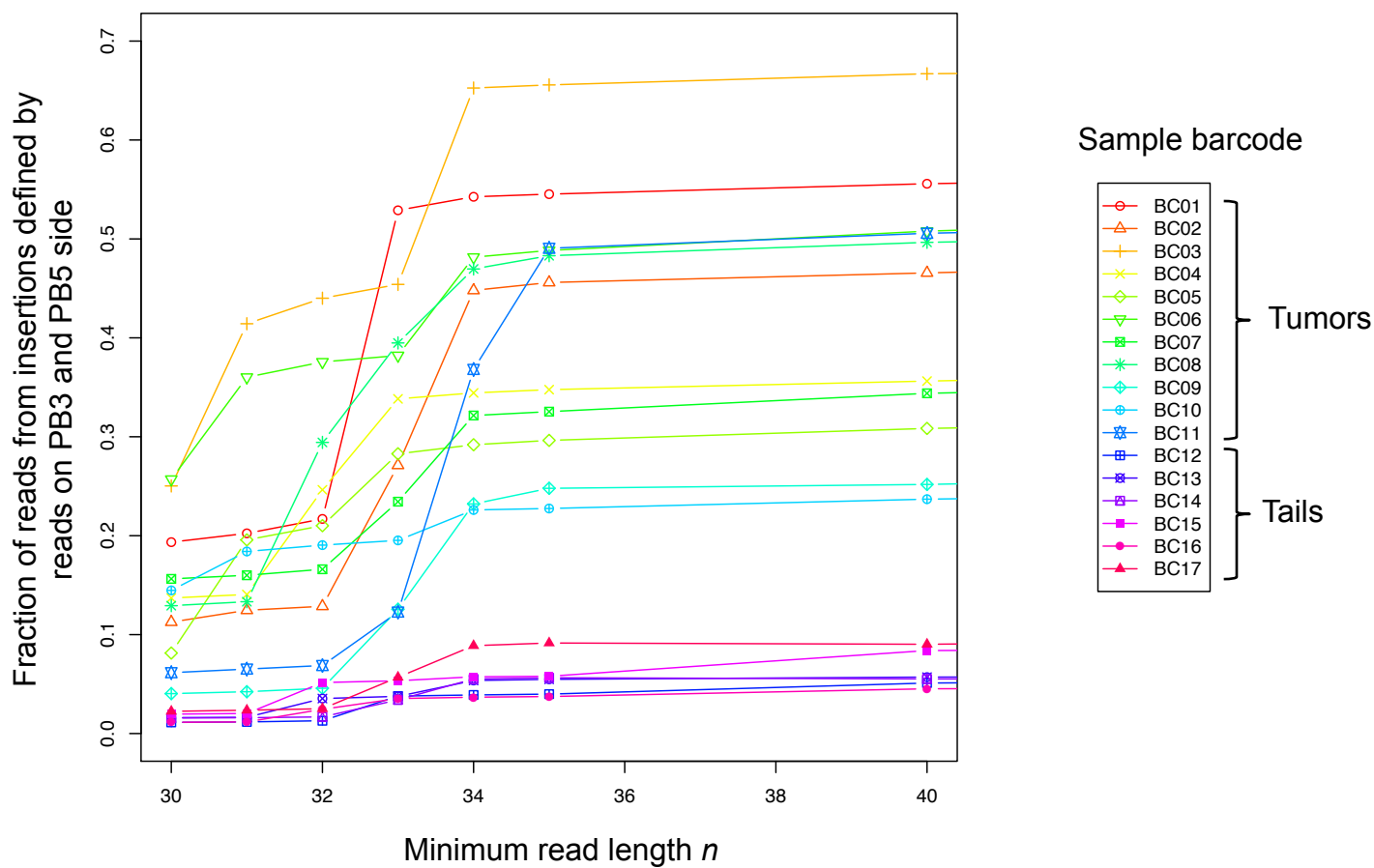

Supplement: Figure S8 — The fraction of reads from insertions with reads on both the PB3 and PB5 sides were plotted against different minimum read length values for identification of insertion sites. A value of n=34 was chosen for final analysis (see Methods for details). Note the significant higher fraction of reads for PB3/PB5 insertions sites in tumor (BC01-BC11) vs. tail samples (BC12-BC17). (PDF) [file pone.0072338.s008.pdf]
