# Supplementary material for: Clonal Expansion Analysis of Transposon Insertions by High-Throughput Sequencing Identifies Candidate Cancer Genes in a PiggyBac Mutagenesis Screen
Source: PLoS One. 2013 Aug 5;8(8):e72338. doi: 10.1371/journal.pone.0072338 (PMC3733837; doi:10.1371/journal.pone.0072338)
Supplement: Figure S6 — A) Two pieces of a Formalin-stored sample of Tumor 05 were dissected and processed independently. Analysis of PiggyBac insertion sites and read frequencies were performed for the PB5 side of the transposon. Box plot diagram of read frequencies and tabular lists of enriched insertions above the 0.37% threshold show strong correlation between the samples. The 6 highest insertions rank in identical order, and more than 50% of enriched insertions are overall identical. B) A lung tumor that had been obtained in a transposon mutagenesis mouse with additional heterozygous background mutation of the tumor suppressor INK4A/Arf [28] was dissected into two pieces that were processed independently for read frequencies from the PB5 side. Box plot diagram of read frequencies and tabular lists of enriched insertions above the 0.37% threshold show strong correlation, with more than 50% identical enriched insertions. (PDF) [file pone.0072338.s006.pdf]

A

Tumor 05, liver<br>Formalin fixed tissue		
	Insertions	PB5 Reads
Tumor 05, A	1857	90406
Tumor 05, B	1902	50160

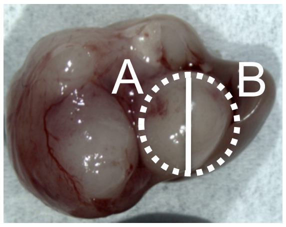

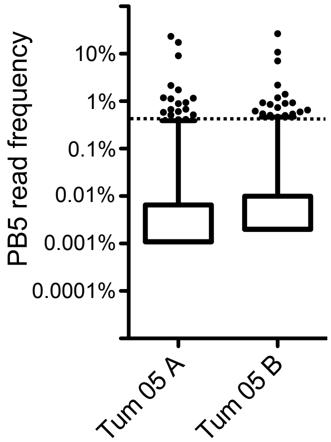

Tumor 05 A

Chr	Position	PB5 f	
chr11	41573884	23.2%	intergenic
chr18	16209166	17.4%	intergenic
chr1	87855454	9.1%	***Dgkd***
chr8	38677940	2.2%	intergenic
chr19	34319719	1.7%	***Fas***
chr19	32786662	1.2%	***Pten***
chr8	100880553	1.2%	intergenic
chr13	69768968	1.1%	intergenic
chr6	120080572	0.9%	intergenic
chr1	156608148	0.9%	***Abl2***
chr3	100720626	0.7%	intergenic
chr9	114777333	0.7%	***Cmtm7***
chr1	181237712	0.6%	intergenic
chr16	23230498	0.6%	***St6gal1***
chr1	134962318	0.5%	intergenic
chr6	131293588	0.5%	***Magohb***
chr13	32829461	0.4%	intergenic
chr2	158268767	0.4%	***Bpi1***
chr9	98589940	0.4%	***Mrps22***

Tumor 05 B

Chr	Position	PB5 f	
chr11	41573884	26.5%	intergenic
chr18	16209166	10.7%	intergenic
chr1	87855454	7.1%	***Dgkd***
chr8	38677940	2.2%	intergenic
chr19	34319719	1.4%	***Fas***
chr19	32786662	1.2%	***Pten***
chr1	156608148	0.9%	***Abl2***
chr1	97039868	0.9%	intergenic
chr6	120080572	0.9%	intergenic
chr9	114777333	0.9%	***Cmtm7***
chr11	22125225	0.7%	***Ehbp1***
chr9	63572479	0.7%	***Iqch***
chr6	131293588	0.6%	***Magohb***
chr8	100880553	0.6%	intergenic
chr13	69768968	0.5%	intergenic
chr16	95849795	0.5%	***1600002D24Rik***
chr9	5699470	0.5%	intergenic
chr9	98589940	0.5%	***Mrps22***
chr2	11637292	0.5%	intergenic
chr8	87954812	0.5%	***Zfp423***
chr3	100720626	0.4%	intergenic
chr8	28543227	0.4%	intergenic
chr12	53713078	0.4%	***Npas3***
chr15	39478446	0.4%	***Rims2***

B

Animal ID #4180<br>R26-PBase; ATP1-S2; INK+/-<br>Age: 42 weeks		
	Insertions	PB5 Reads
Lung tumor, A	1327	16314
Lung tumor, B	1109	11394

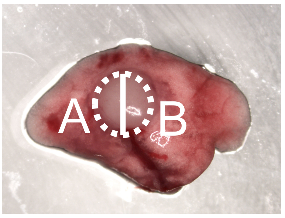

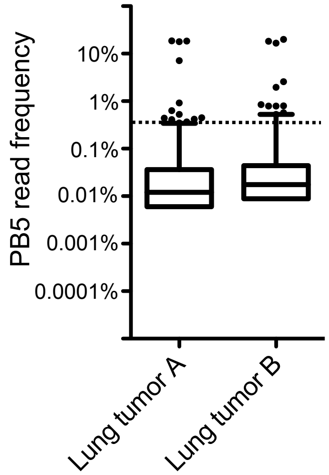

Lung tumor A

Chr	Position	PB5 f	
chr11	80623112	18.6%	***Myo1d***
chr9	89968709	18.4%	***Rasgrf1***
chr12	69422766	17.8%	intergenic
chr10	56904704	7.1%	intergenic
chr18	14015898	0.9%	intergenic
chr10	15213909	0.6%	intergenic
chr10	32264523	0.5%	***Nkain2***
chr10	67016841	0.4%	***Reep3***
chr10	86146164	0.4%	***Syn3***
chr6	17017596	0.4%	intergenic
chr4	54976180	0.4%	***Zfp462***

Lung tumor B

Chr	Position	PB5 f	
chr12	69422766	19.9%	intergenic
chr11	80623112	18.2%	***Myo1d***
chr9	89968709	16.6%	***Rasgrf1***
chr10	56904704	2.6%	intergenic
chr7	143292394	2.0%	***Kcnq1***
chr2	52623038	0.8%	***Cacnb4***
chr18	14015898	0.8%	intergenic
chr10	15061726	0.8%	intergenic
chrX	89274343	0.8%	intergenic
chr10	86146164	0.6%	***Syn3***
chr2	76150368	0.5%	***Pde11a***
chr10	119901129	0.5%	***Grip1***
chr10	32264523	0.5%	***Nkain2***
chr10	67016841	0.5%	***Reep3***
chr2	49096760	0.4%	***Mbd5***
chr10	15091000	0.4%	intergenic
