# Supplementary material for: Clonal Expansion Analysis of Transposon Insertions by High-Throughput Sequencing Identifies Candidate Cancer Genes in a PiggyBac Mutagenesis Screen
Source: PLoS One. 2013 Aug 5;8(8):e72338. doi: 10.1371/journal.pone.0072338 (PMC3733837; doi:10.1371/journal.pone.0072338)

A

Animal ID #4180<br>R26-PBase; ATP1-S2; INK+/-<br>Age: 42 weeks		
	Insertions	Reads
Lung tumor, A	1327	16314
Lung, adjacent	2998	28685
Liver	4262	50388
Tail	4675	54957

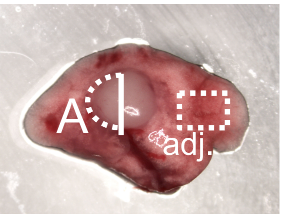

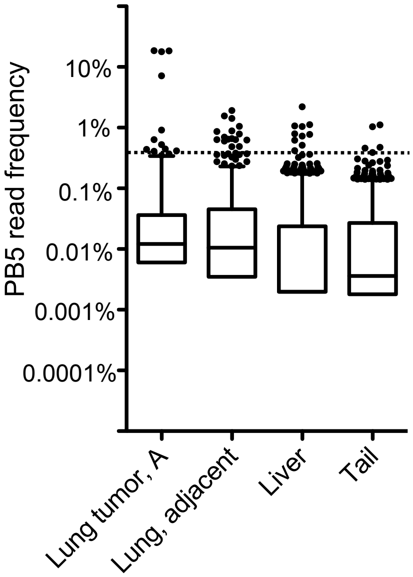

Expanded insertions into genes

#4180	
Lung tumor, A	
Gene	*PB5 f*
Myo1d	18.6%
Rasgrf1	18.4%
Nkain2	0.5%
Reep3	0.4%
Syn3	0.4%
Zfp462	0.4%

#4180	
Lung, adjacent	
Gene	*PB5 f*
Myo1d	1.9%
Gstt1	1.6%
Reep3	1.4%
Pcolce2	1.0%
Pde11a	0.7%
Kif12	0.7%
Syn3	0.6%
Rgs6	0.5%
Ablim1	0.5%
Hs3st5	0.4%
Pcyt1b	0.4%

#4180	
Liver	
Gene	*PB5 f*
Myo1d	1.1%
Spice1	0.8%
Gstt1	0.8%

#4180	
Tail	
Gene	*PB5 f*
Myo1d	1.1%
Ano3	1.0%
1700020G	0.4%
Nuggc	0.3%
Zbtb16	0.3%

B

Animal ID #3805<br>R26-PBase; ATP1-S2<br>Age: 58 weeks		
	Insertions	PB5 Reads
Heart	3378	25599
Kidney	7680	143862
Liver	8690	131204
Lung	3507	39058
Spleen	4379	58661
Tail	4145	25855

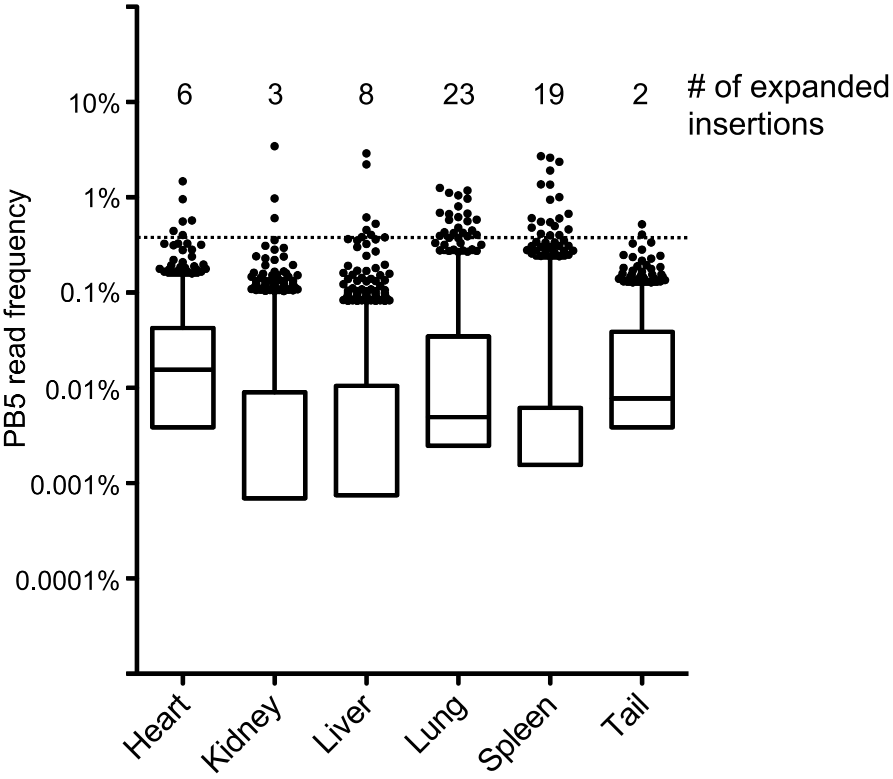

Supplement: Figure S7 — A) Transposon insertions in a lung tumor that had been obtained in a transposon mouse with INK4A/Arf+/- background were analyzed for PB5 side read frequencies and compared to samples from adjacent normal lung tissue, liver, and tail of the same mouse. The tabular listings of enriched insertions in genes in these samples reveal that the lung tumor carries an expanded insertion in the Rasrgf1 gene, which was not obtained in other tissues. Rasgrf1 is therefore a strong candidate cancer gene. Of note, Rasgrf1 is upregulated in lung tumors [29], and Rasgrf1 is also the most enriched insertion site in the lung Tumor 06 (see Figure 4A). An expanded insertion in the Myod1 gene was also observed the tumor sample, which was found at about tenfold lower frequency in other tissues and tail. Expanded insertions in the Reep3 and Syn3 genes were found both in tumor and adjacent normal lung tissue at similar frequency, suggesting that these insertions are already present in normal tissue and are not tumor driving. B) The tumor free transposon mutagenesis mouse #3805 was dissected, and PiggyBac insertion profiles of different organs and tails were compared. The number of expanded insertions above the 0.37% threshold is indicated in the diagram. Between 3 and 23 enriched insertions were found in organs, and 2 in tail tissue. (PDF) [file pone.0072338.s007.pdf]
