# Supplementary material for: Clonal Expansion Analysis of Transposon Insertions by High-Throughput Sequencing Identifies Candidate Cancer Genes in a PiggyBac Mutagenesis Screen
Source: PLoS One. 2013 Aug 5;8(8):e72338. doi: 10.1371/journal.pone.0072338 (PMC3733837; doi:10.1371/journal.pone.0072338)
Supplement: Methods S1 — (PDF) [file pone.0072338.s012.pdf]

Supplementary Methods

Splinkerette with acoustic shearing for preparation of transposon insertion site libraries for Illumina sequencing

Genomic DNA was isolated from tumor samples by overnight digestion in lysis buffer (0.1 M Tris pH 8.5, 0.2 M NaCl, 5 mM EDTA, 0.2% SDS, 0.1 mg/ml Proteinase K) at 55°C, followed by 2-propanol precipitation, 70% ethanol wash, and resuspension overnight in TE buffer (10mM Tris, 1 mM EDTA, pH 7.4) at 55°C.

To shear DNA into fragments of 200 – 400 bp size, 3 µg of DNA in a volume of 120 µl TE were sonicated in a Covaris E210 acoustic focusing instrument (parameter settings: Time: 600 sec, Duty Cycle: 5%, Intensity: 2, Cycles per burst: 50). DNA was purified with a Qiaquick spin column (QIAGEN) and eluted in a volume of 30 µl EB buffer. For quality control, 5 µl of the eluted DNA was analyzed on a 0.8% agarose gel.

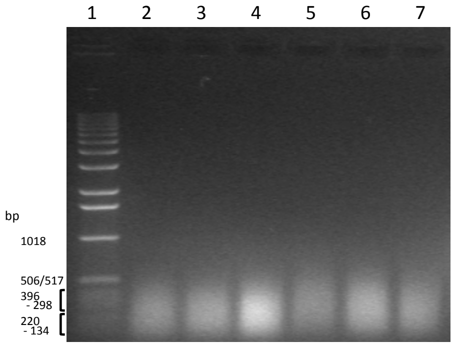

Example of sheared DNA separated on 0.8% agarose gel

Lane 1: 1 kb ladder, Invitrogen

Lane 2-7: DNA sheared with Covaris E210 sonicator

DNA Fragment End repair

14.5 µl	Qiaquick purified genomic DNA, sheared by Covaris sonication
2 µl	10x T4 DNA ligase buffer w/ 10 mM ATP (NEB)
1 µl	dNTP (10 mM)
1 µl	T4 DNA Polymerase
1 µl	Klenow DNA Polymerase
0.5 µl	T4 Polynucleotid kinase
---	
20 µl	

30 min incubation at RT

DNA was purified with a Qiaquick spin column and eluted in 42 µl EB.

A-tailing of DNA fragments

42 µl Qiaquick purified end-repaired DNA
1 µl dATP (10 mM)
5 µl 10x PCR buffer ThermoPol (NEB)
2 µl Taq
---
50 µl

15 min incubation at 72°C
The entire reaction was EtOH precipitated by using 2 µg Glycogen as carrier and resuspended in 7.5 µl H_2O.

Preparing Splinkerette adaptor

(final adaptor conc. 25 µM)
Mix:
25 µl Adaptor oligo "plus" strand (100 µM)
25 µl Adaptor oligo "minus" strand (100 µM)
20 µl NEB restriction buffer 2
30 µl H_2O
Boil for 2 min, let cool down at RT. Store at -20°C.

Ligation of Splinkerette adaptor

7.5 µl A-tailed DNA fragments
0.5 µl Adaptor SPLK-T (25 µM)
1 µl 10x T4 Ligase buffer
1 µl T4 Ligase
--
10 µl

Ligation was performed o/n at 16°C
The ligation was purified with a Qiaquick spin column and eluted in 40 µl EB.

First Splinkerette PCR

2.5 µl Qiaquick purified ligation product
5 µl 5x Phusion HF buffer
0.5 µl dNTP (10 mM)
0.5 µl Sp-1 (10µM)
0.5 µl PB5-1 (10µM)
0.25 µl Phusion enzyme
15.75 µl H_2O

25µl

3 min 95°C
35 cycles: 15 sec 95°C, 30 sec 65°C, 30 sec 72°C

Second Splinkerette PCR

A 1:200 dilution of the first PCR was prepared with H_2O

2.5µl 1:200 dilution of first PCR
5 µl 5x Phusion HF buffer
0.5 µl dNTP (10 mM)
0.5 µl P7-Sp-2 (10µM)
0.5 µl P5-XX-PB5-2 (10µM) (XX = barcode number)
0.25 µl Phusion enzyme
15.75 µl H_2O

25µl

3 min 95°C
35 cycles: 15 sec 95°C, 30 sec 60°C, 30 sec 72°C

12.5 µl of the PCR product was separated on a 0.8% agarose gel
PCR product was cut out in a size range of 200 to 400 bp size, purified with a Qiaquick spin column and eluted in 30 µl EB.

DNA concentration was determined with a Nanodrop device. To reach a final concentration of the pooled sample of >30 ng/µl in 20 µl H_2O, appropriate amounts of all samples were pooled together, EtOH precipitated, and resuspended in 20 µl H_2O, and submitted to the sequencing facility at Mount Sinai School of Medicine. Samples were mixed with 50% PhiX Genomic DNA library (Illumina) to increase cluster diversity on the Illumina chip. Sample pools were sequenced on a single lane of an Illumina HiSeq 2000 Instrument as single unpaired reads with a length of 100 bases.

Structure of final Splinkerette PCR product

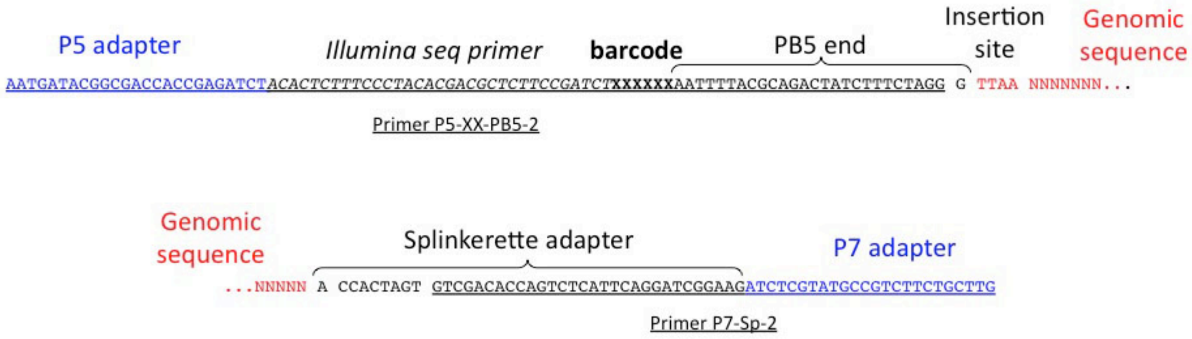

Structure of PB5 insertion site library is shown, PB3 library has same structure, except PB3 end part (see Oligonucleotide Sequences for details).

Oligonucleotide Sequences

Oligonucleotide sequences			
**Purpose**	**Name**	**Sequence 5' - 3'**	**Description**
Splinkerette linker	SplkTp	CGAAGAGTAACCGTTGCTAGGAGAGACCGTGGCTGAATGAGACTGGTGTCGACACTAGTGG*T	Splinkerette T-overhang linker, plus strand (* = phosphorothioate bond)
	SplkTm	(5'phospho)CCACTAGTGTCGACACCAGTCTCTAATTTTTTTTTTCAAAAAAA	Splinkerette T-overhang linker, minus strand
First round Splinkerette PCR	Sp-1	GAGTAACCGTTGCTAGGAGAG	Primer on Splinkerette linker
	PB3-1	CGCATGATTATCTTTAACGTACGTCAC	Primer on PB3 end
	PB5-1	CAAGAATGCATGCGTCAATTTTACGC	Primer on PB5 end
Second round Splinkerette PCR	P7-Sp-2	CAAGCAGAAGACGGCATACGAGATCTTCCGATCCTGAATGAGACTGGTGTCGAC	Illumina P7 adapter + primer on Splinkerette linker
	P5-01-PB5-2	AATGATACGGCGACCACCGAGATCT*ACACTCTTTCCCTACACGACGCTCTTCCGATCT***ATCACG**AATTTTACGCAGACTATCTTTCTAGG	Illumina P5 adapter + *sequencing primer site* + **barcode 01** + primer on PB5 end
	P5-02-PB5-2	AATGATACGGCGACCACCGAGATCT*ACACTCTTTCCCTACACGACGCTCTTCCGATCT***CGATGT**AATTTTACGCAGACTATCTTTCTAGG	Illumina P5 adapter + *sequencing primer site* + **barcode 02** + primer on PB5 end
	P5-03-PB5-2	AATGATACGGCGACCACCGAGATCT*ACACTCTTTCCCTACACGACGCTCTTCCGATCT***TTAGGC**AATTTTACGCAGACTATCTTTCTAGG	Illumina P5 adapter + *sequencing primer site* + **barcode 03** + primer on PB5 end
	P5-04-PB5-2	AATGATACGGCGACCACCGAGATCT*ACACTCTTTCCCTACACGACGCTCTTCCGATCT***TGACCA**AATTTTACGCAGACTATCTTTCTAGG	Illumina P5 adapter + *sequencing primer site* + **barcode 04** + primer on PB5 end
	P5-05-PB5-2	AATGATACGGCGACCACCGAGATCT*ACACTCTTTCCCTACACGACGCTCTTCCGATCT***ACATGT**AATTTTACGCAGACTATCTTTCTAGG	Illumina P5 adapter + *sequencing primer site* + **barcode 05** + primer on PB5 end
	P5-06-PB5-2	AATGATACGGCGACCACCGAGATCT*ACACTCTTTCCCTACACGACGCTCTTCCGATCT***GCCAAT**AATTTTACGCAGACTATCTTTCTAGG	Illumina P5 adapter + *sequencing primer site* + **barcode 06** + primer on PB5 end
	P5-07-PB5-2	AATGATACGGCGACCACCGAGATCT*ACACTCTTTCCCTACACGACGCTCTTCCGATCT***CAGATC**AATTTTACGCAGACTATCTTTCTAGG	Illumina P5 adapter + *sequencing primer site* + **barcode 07** + primer on PB5 end
	P5-08-PB5-2	AATGATACGGCGACCACCGAGATCT*ACACTCTTTCCCTACACGACGCTCTTCCGATCT***ACTTGA**AATTTTACGCAGACTATCTTTCTAGG	Illumina P5 adapter + *sequencing primer site* + **barcode 08** + primer on PB5 end
	P5-09-PB5-2	AATGATACGGCGACCACCGAGATCT*ACACTCTTTCCCTACACGACGCTCTTCCGATCT***GATCAG**AATTTTACGCAGACTATCTTTCTAGG	Illumina P5 adapter + *sequencing primer site* + **barcode 09** + primer on PB5 end
	P5-10-PB5-2	AATGATACGGCGACCACCGAGATCT*ACACTCTTTCCCTACACGACGCTCTTCCGATCT***TAGCTT**AATTTTACGCAGACTATCTTTCTAGG	Illumina P5 adapter + *sequencing primer site* + **barcode 10** + primer on PB5 end
	P5-11-PB5-2	AATGATACGGCGACCACCGAGATCT*ACACTCTTTCCCTACACGACGCTCTTCCGATCT***GGCTAG**AATTTTACGCAGACTATCTTTCTAGG	Illumina P5 adapter + *sequencing primer site* + **barcode 11** + primer on PB5 end
	P5-12-PB5-2	AATGATACGGCGACCACCGAGATCT*ACACTCTTTCCCTACACGACGCTCTTCCGATCT***CTTGTA**AATTTTACGCAGACTATCTTTCTAGG	Illumina P5 adapter + *sequencing primer site* + **barcode 12** + primer on PB5 end
	P5-13-PB5-2	AATGATACGGCGACCACCGAGATCT*ACACTCTTTCCCTACACGACGCTCTTCCGATCT***CGTGAT**AATTTTACGCAGACTATCTTTCTAGG	Illumina P5 adapter + *sequencing primer site* + **barcode 13** + primer on PB5 end
	P5-14-PB5-2	AATGATACGGCGACCACCGAGATCT*ACACTCTTTCCCTACACGACGCTCTTCCGATCT***ACATCG**AATTTTACGCAGACTATCTTTCTAGG	Illumina P5 adapter + *sequencing primer site* + **barcode 14** + primer on PB5 end
	P5-15-PB5-2	AATGATACGGCGACCACCGAGATCT*ACACTCTTTCCCTACACGACGCTCTTCCGATCT***GCCTAA**AATTTTACGCAGACTATCTTTCTAGG	Illumina P5 adapter + *sequencing primer site* + **barcode 15** + primer on PB5 end
	P5-16-PB5-2	AATGATACGGCGACCACCGAGATCT*ACACTCTTTCCCTACACGACGCTCTTCCGATCT***TGGTCA**AATTTTACGCAGACTATCTTTCTAGG	Illumina P5 adapter + *sequencing primer site* + **barcode 16** + primer on PB5 end
	P5-17-PB5-2	AATGATACGGCGACCACCGAGATCT*ACACTCTTTCCCTACACGACGCTCTTCCGATCT***CACTGT**AATTTTACGCAGACTATCTTTCTAGG	Illumina P5 adapter + *sequencing primer site* + **barcode 17** + primer on PB5 end
	P5-01-PB3-2	AATGATACGGCGACCACCGAGATCT*ACACTCTTTCCCTACACGACGCTCTTCCGATCT***ATCACG**CGTCACAATATGATTATCTTTCTAGG	Illumina P5 adapter + *sequencing primer site* + **barcode 01** + primer on PB3 end
	P5-02-PB3-2	AATGATACGGCGACCACCGAGATCT*ACACTCTTTCCCTACACGACGCTCTTCCGATCT***CGATGT**CGTCACAATATGATTATCTTTCTAGG	Illumina P5 adapter + *sequencing primer site* + **barcode 02** + primer on PB3 end
	P5-03-PB3-2	AATGATACGGCGACCACCGAGATCT*ACACTCTTTCCCTACACGACGCTCTTCCGATCT***TTAGGC**CGTCACAATATGATTATCTTTCTAGG	Illumina P5 adapter + *sequencing primer site* + **barcode 03** + primer on PB3 end
	P5-04-PB3-2	AATGATACGGCGACCACCGAGATCT*ACACTCTTTCCCTACACGACGCTCTTCCGATCT***TGACCA**CGTCACAATATGATTATCTTTCTAGG	Illumina P5 adapter + *sequencing primer site* + **barcode 04** + primer on PB3 end
	P5-05-PB3-2	AATGATACGGCGACCACCGAGATCT*ACACTCTTTCCCTACACGACGCTCTTCCGATCT***ACATGT**CGTCACAATATGATTATCTTTCTAGG	Illumina P5 adapter + *sequencing primer site* + **barcode 05** + primer on PB3 end
	P5-06-PB3-2	AATGATACGGCGACCACCGAGATCT*ACACTCTTTCCCTACACGACGCTCTTCCGATCT***GCCAAT**CGTCACAATATGATTATCTTTCTAGG	Illumina P5 adapter + *sequencing primer site* + **barcode 06** + primer on PB3 end
	P5-07-PB3-2	AATGATACGGCGACCACCGAGATCT*ACACTCTTTCCCTACACGACGCTCTTCCGATCT***CAGATC**CGTCACAATATGATTATCTTTCTAGG	Illumina P5 adapter + *sequencing primer site* + **barcode 07** + primer on PB3 end
	P5-08-PB3-2	AATGATACGGCGACCACCGAGATCT*ACACTCTTTCCCTACACGACGCTCTTCCGATCT***ACTTGA**CGTCACAATATGATTATCTTTCTAGG	Illumina P5 adapter + *sequencing primer site* + **barcode 08** + primer on PB3 end
	P5-09-PB3-2	AATGATACGGCGACCACCGAGATCT*ACACTCTTTCCCTACACGACGCTCTTCCGATCT***GATCAG**CGTCACAATATGATTATCTTTCTAGG	Illumina P5 adapter + *sequencing primer site* + **barcode 09** + primer on PB3 end
	P5-10-PB3-2	AATGATACGGCGACCACCGAGATCT*ACACTCTTTCCCTACACGACGCTCTTCCGATCT***TAGCTT**CGTCACAATATGATTATCTTTCTAGG	Illumina P5 adapter + *sequencing primer site* + **barcode 10** + primer on PB3 end
	P5-11-PB3-2	AATGATACGGCGACCACCGAGATCT*ACACTCTTTCCCTACACGACGCTCTTCCGATCT***GGCTAG**CGTCACAATATGATTATCTTTCTAGG	Illumina P5 adapter + *sequencing primer site* + **barcode 11** + primer on PB3 end
	P5-12-PB3-2	AATGATACGGCGACCACCGAGATCT*ACACTCTTTCCCTACACGACGCTCTTCCGATCT***CTTGTA**CGTCACAATATGATTATCTTTCTAGG	Illumina P5 adapter + *sequencing primer site* + **barcode 12** + primer on PB3 end
	P5-13-PB3-2	AATGATACGGCGACCACCGAGATCT*ACACTCTTTCCCTACACGACGCTCTTCCGATCT***CGTGAT**CGTCACAATATGATTATCTTTCTAGG	Illumina P5 adapter + *sequencing primer site* + **barcode 13** + primer on PB3 end
	P5-14-PB3-2	AATGATACGGCGACCACCGAGATCT*ACACTCTTTCCCTACACGACGCTCTTCCGATCT***ACATCG**CGTCACAATATGATTATCTTTCTAGG	Illumina P5 adapter + *sequencing primer site* + **barcode 14** + primer on PB3 end
	P5-15-PB3-2	AATGATACGGCGACCACCGAGATCT*ACACTCTTTCCCTACACGACGCTCTTCCGATCT***GCCTAA**CGTCACAATATGATTATCTTTCTAGG	Illumina P5 adapter + *sequencing primer site* + **barcode 15** + primer on PB3 end
	P5-16-PB3-2	AATGATACGGCGACCACCGAGATCT*ACACTCTTTCCCTACACGACGCTCTTCCGATCT***TGGTCA**CGTCACAATATGATTATCTTTCTAGG	Illumina P5 adapter + *sequencing primer site* + **barcode 16** + primer on PB3 end
	P5-17-PB3-2	AATGATACGGCGACCACCGAGATCT*ACACTCTTTCCCTACACGACGCTCTTCCGATCT***CACTGT**CGTCACAATATGATTATCTTTCTAGG	Illumina P5 adapter + *sequencing primer site* + **barcode 17** + primer on PB3 end
